# Butterflies as bioindicators of metal contamination

**DOI:** 10.1007/s11356-023-28930-x

**Published:** 2023-08-08

**Authors:** Matteo Pallottini, Enzo Goretti, Chiara Argenti, Gianandrea La Porta, Laura Tositti, Enrico Dinelli, Beatrice Moroni, Chiara Petroselli, Paola Gravina, Roberta Selvaggi, David Cappelletti

**Affiliations:** 1 Department of Chemistry, Biology and Biotechnology, University of Perugia, 06123 Perugia, PG Italy; 2 Department of Chemistry “G. Ciamician”, University of Bologna, 40126 Bologna, BO Italy; 3 Department of Biological, Geological and Environmental Sciences, University of Bologna, 40126 Bologna, BO Italy

**Keywords:** Butterflies, Contamination, Trace metals, Biomonitoring

## Abstract

Anthropogenic trace metal contamination has significantly increased and has caused many hazardous consequences for the ecosystems and human health. The Terni basin valley (Central Italy) shows a heavy load of pollutants from industrial activities, while the characteristic orography structure of the valley favours air stagnation, thus limiting air pollution dispersal. The present study conducted in 2014 aimed to determine the concentration of ten metals in five species of butterflies at nine sites in the Terni valley along a 21-km-long transect, including both relatively pristine and industrial areas. At sites where soil contamination was high for a given metal, such as for chromium as in the case of site 4 (the closest to the steel plant) and for lead as in the case of site 2 (contaminated by a firing range), higher levels of contamination were observed in the tissues of butterflies. We found a correlation between soil contamination and the concentration of Cr, Al and Sr in the tissues of some species of butterflies. The sensitivity to contamination differed among the five species; in particular, *Coenonympha pamphilus* was generally the species that revealed the highest concentrations of all the ten trace metals at the sites closer to the industrial area. It is known that *C. pamphilus* is a sedentary species and that its host plants are the Poaceae, capable of accumulating high quantities of metals in their rhizosphere region, thus providing the link with soil contamination. Therefore, monitoring the metal concentration levels in butterflies might be a good indicator and a control tool of environmental quality, specifically in areas affected by high anthropogenic pollution loads linked to a specific source.

**Supplementary Information:**

The online version contains supplementary material available at 10.1007/s11356-023-28930-x.

## Introduction

In the last decades, technological and industrial development has led to the widespread dispersal of anthropogenic trace elements with a significant impact on ecosystems and human health (Järup 2003; WHO 2014; Pallottini et al. 2017a). Their impact on the biosphere is tightly associated with source emission, recirculation pathway, chemical speciation and bioavailability (Vet et al. 2014a,b). Metals such as cadmium and lead are toxic even at low concentrations, as they are not part of any useful metabolic functions. In contrast, aluminium, manganese, iron, chromium, nickel, copper, zinc and strontium are essential for biota; however, they can become toxic at concentrations above specific threshold levels (WHO 1996; Tchounwou et al. 2012; ATSDR 2019).

Detection and quantification of trace element contamination in the environment are always challenging (Selvaggi et al. 2020). Water distributes contaminants fairly rapidly across a wide area in aquatic ecosystems, eventually depositing them onto bottom sediments (Goretti et al. 2016; Miranda et al. 2021). On the other side, in the terrestrial environment, we observe considerable interferences in detecting contamination due to the lithogenic components rich in inorganic components. Moreover, thanks to post-depositional modifications mainly in association with hydrometeors, the overall effect is the masking of exogenous pollution sources, such as the influence of atmospheric pollutants like airborne particulate. Therefore, the use of bioindicators as sentinel species (Beeby 2001) is a primary tool for adequate monitoring of terrestrial ecosystems, wherein contaminants are usually localized and not diffused in the environment (Wuana and Okieimen 2011; Gall et al. 2015; Goretti et al. 2018).

Insects are invertebrates often used as bioindicators for metal pollution (Nummelin et al. 2007; Azam et al. 2015; Monchanin et al. 2021). Many studies have attempted to interpret metal accumulation in insects from contaminated and less polluted ecosystems, both aquatic and terrestrial (Guimarães Souto et al. 2019; Irfan Dar et al. 2019). Only a few studies have been carried out on Lepidoptera, though they have an ecological relevance similar to the deeply studied honey bees, owing to their pollinating role. Moreover, unlike bees, many species of butterflies live in even more limited territories and could therefore act as efficient detectors of local environmental contamination. While the occurrence of Lepidoptera has been long associated with their role as bioindicators (e.g. climate change), their role as detectors of metal contamination seems to be limitedly defined and scarcely applied (Fleishman and Murphy 2009).

Most of the studies on metals in Lepidoptera are mainly focused on species of economic interests, such as agricultural pests (*Spodoptera litura* (Fabricius, 1775)), defoliators (*Lymantria dispar* (Linnaeus, 1758)) and silk producers (*Bombyx mori* (Linnaeus, 1758)).

The studies are usually based on laboratory exposure tests in which physiological or behavioural responses to trace metal exposure are assessed (Bischof 1995, 1996; Wang et al. 2004; Mirčić et al. 2013; Ali et al. 2019), or in which the accumulation levels are evaluated (Prince et al. 2001; Ashfaq et al. 2009, 2010; Zhuang et al. 2009; Zhou et al. 2012, 2015a; Vlahović et al. 2017; Zhang et al. 2020; Monchanin et al. 2021).

On the other hand, there are fewer investigations based on field studies. Some researchers consider the relationship between the butterfly bioindicator species occurrence and environmental contamination level (Mulder et al. 2005; Mulder and Breure 2006; Salz and Fartmann 2017). Others evaluated the concentration in butterfly tissues relating them with the levels of trace metals in the environment (Heliövaara et al. 1987; van San and Spitzer 1993; Naumova et al. 1994; Bagatto and Shorthouse 1996; Kozlov et al. 2000; Azam et al. 2015; Lin et al. 2019; Perić-Mataruga et al. 2019). Even fewer articles report about the simultaneous observations among different species (Heliövaara and Väisänen 1990; Naumova et al. 1994), and even those that focus only on adult specimens are rare (van San and Spitzer 1993; Azam et al. 2015; Lin et al. 2019). Variable accumulation levels in butterflies have been observed likely as a function of the different trace elements (Naumova et al. 1994; Bagatto and Shorthouse 1996; Kozlov et al. 2000; Prince et al. 2001; Lin et al. 2019).

The main findings of these studies are that (i) the biological stages of the lepidopteran life cycle (from egg to adult) often show different values of metal accumulation (Bagatto and Shorthouse 1996; Zhou et al. 2015b; Jin et al. 2020); (ii) species-specific differences are found (Heliövaara and Väisänen 1990); (iii) element-specific accumulation levels are possible (Naumova et al. 1994; Bagatto and Shorthouse 1996; Kozlov et al. 2000; Prince et al. 2001; Lin et al. 2019); (iv) associations between metal accumulation and different matrices are found (e.g. soil, larval host plants) (Heliövaara and Väisänen 1990, Bagatto and Shorthouse 1996; Zhuang et al. 2009; Ding et al. 2013; Zhou et al. 2015a; Eeva et al. 2018; Lin et al. 2019 in soil). In particular, some authors pointed out a clear response of Lepidoptera metal accumulation levels to an environmental pollution gradient (Heliövaara and Väisänen 1990; Bagatto and Shorthouse 1996; Kozlov et al. 2000; Eeva et al. 2018).

The present paper focuses on the use of adult butterflies (order Lepidoptera, superfamily Papilionoidea) as bioindicators of trace metal levels, relating the concentration in their tissue with environmental contamination. We additionally propose using different butterfly species occurring in the study area. The choice of butterflies as environmental quality bioindicators is based on several factors. Many common species of butterflies are present in both natural, semi-natural and anthropized habitats, representing regional biodiversity (Wang et al. 2017). They are fairly easy to sample and identify (Qian Tan et al. 2018); as flying insects, they can explore territories intensively, visiting numerous flowers and thus intercepting contaminants and pollutants present in the ecosystem (Corke 1999). They are also intimately linked to the host plants at the larval stage (Bagatto and Shorthouse 1996; Kozlov et al. 2000; Mulder et al. 2005; Mulder and Breure 2006). Finally, it is emblematic how some charismatic species have significant social and media relevance for their beauty (Habel et al. 2021) while simultaneously accounting for many endangered species (Balletto et al. 2015; Bonelli et al. 2018).

The present study has been carried out across the Terni valley (Central Italy), a current hot spot for environmental studies, due to its heavy load of pollutants by industrial activities further promoted by its orographic conditions favouring air stagnation and pollutant accumulation (Ferrero et al. 2012, 2014; Moroni et al. 2012, 2013; Tositti et al. 2020). In the present study, we focus on butterflies and their potential sensitivity to reflect in their tissues the trace element ambient concentrations.

Specifically, the study aims to evaluate the trace metal contamination of different butterfly species collected along the transect mentioned above at increasing distances from the Terni urban-industrial area, in comparison with soil contamination, and thus obtain an integrated picture of the overall environmental conditions across the investigated district.

## Materials and methods

### Study area

**Fig. 1:**
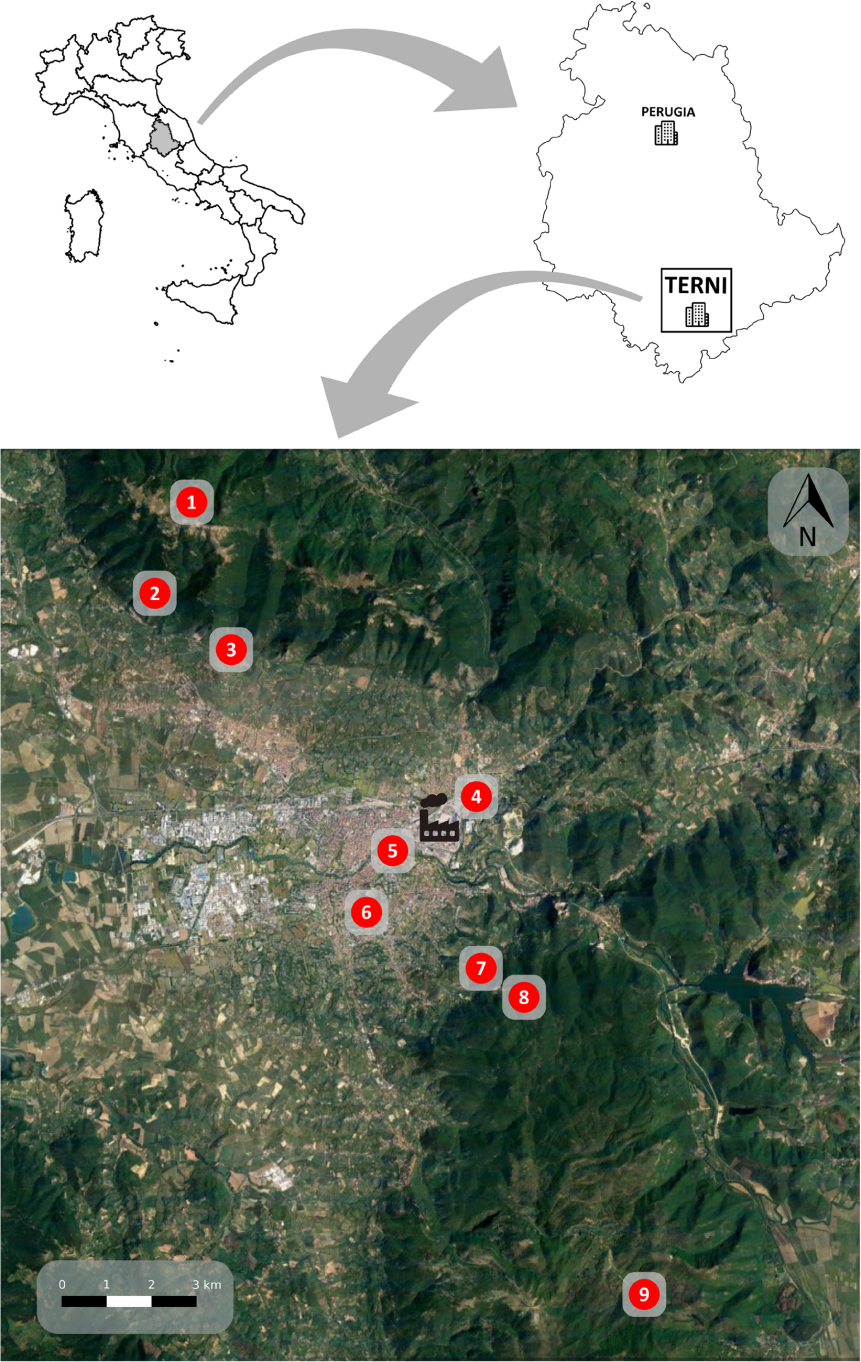
Study area: nine sampling sites and the AST (Acciai Speciali Terni) steel plant in the Terni basin (Umbria Region, Italy)

The survey spanned over the whole Terni basin valley (Umbria, Central Italy), an area densely populated (about 500 inhabitants km^−2^, ISTAT 2022) and industrialized, located at the margins of the Italian Apennines Mountain range. Terni hosts important European industrial complexes (steel and chemicals production) since the end of the nineteenth century. Therefore, the persistence of pollutants in the survey area is caused by a combination of production plant emissions and a characteristic orographic structure of the valley, which have permanently impacted the ecosystems and even the chemistry of the local natural soils (Ferrero et al. 2012, 2014; Moroni et al. 2012, 2013; Tositti et al. 2020). Recently, Tositti et al. (2020) have investigated the atmospheric deposition processes based on surface soils collected along a transect in the complex mountain orography of the Terni valley. Airborne radiotracers have been used to characterize the atmospheric deposition, and a complete dataset of radioactive and stable trace elements was determined. Many elements of anthropogenic sources showed marked heterogeneity along the transect in association with land use and range/distribution of atmospheric emissions. The monitoring campaign was carried out at nine sites along a transect (NW–SE axis, 21 km long) focused on the city of Terni. Three sites (4: Prisciano, 156 m a.s.l. (above sea level); 5: Pineta Centurini, 133 m a.s.l.; 6: Parco Le Grazie, 149 m a.s.l.) were located close to the primary pollution sources (Acciai Speciali Terni, AST) within the urban territory (1, 1.1 and 2.4 km from the main pollution source, respectively). Six sites were located respectively north (1: Monte Torre Maggiore, 967 m a.s.l.; 2: Sant’Erasmo, 724 m a.s.l.; 3: Cesi, 338 m a.s.l.) and south (7: Larviano, 385 m a.s.l.; 8: Miranda, 618 m a.s.l.; 9: Stroncone, 956 m a.s.l.) from the urban-industrial area, with an increasing distance and elevation from the AST (north: 9.5, 8.5 and 6.5 km from the AST, respectively; south: 3.2, 4.1 and 11.2 km from the AST, respectively). Sites at higher altitudes and at the extremes of the transect in both directions (1 and 9) are located inside protected areas of the Natura 2000 Network (IT5220013 — Monte Torre Maggiore (Monti Martani) and IT5220021 — Piani di Ruschio, respectively) (EEA 2021) (Fig. 1).

### Butterfly sample collection

Butterflies were collected during 17 sampling sessions, conducted at each of the nine sites along the transect, with a comparable sampling effort, between June and September 2014.

Five species of butterflies (order Lepidoptera, superfamily Papilionoidea), belonging to three different families, were sampled: two species of Pieridae: *Pieris napi* (Linnaeus, 1758) and *Pieris rapae* (Linnaeus, 1758); two species of Nymphalidae: *Coenonympha pamphilus* (Linnaeus, 1758) and *Lasiommata megera* (Linnaeus, 1767) and one species of Lycaenidae: *Polyommatus icarus* (Rottemburg, 1775).

These species were selected because of their prevalence in the study area, because they have a usually polyvoltinous (at least bivoltinous) life cycle, and because of their polyphagous larvae (feeding mainly on grass species belonging to Poaceae, Fabaceae and Brassicaceae) (IBC 2009; Pivotti et al. 2011; Tolman and Lewington 2014; Mazzei et al. 2021). Some of these species are strictly confined in small territories, such as *C. pamphilus* and *L. megera*, showing a tendency to poor mobility, in particular *C. pamphilus* (Cormont et al. 2011). *Polyommatus icarus* and *Pieris napi* are able to disperse, but moderately. *Pieris rapae*, on the contrary, is a wandering species that can disperse at great distances through migration. All the biological and ecological features of the five species are reported in the supplementary material. The status of the Italian populations of these five species was classified as least concern (LC), which is adopted for the populations that are not at risk of extinction in the short or medium term (Balletto et al. 2015; Bonelli et al. 2018).

An entomological net (35 cm diameter) made up of fine meshes was used to collect specimens to minimize the damage to the butterflies. The species were identified through butterfly-watching, that is, the observation of the specimens during the flight. All the samples were brought to the laboratory, stored at − 20 °C, and a more accurate identification was further conducted with the help of taxonomic keys (IBC 2009; Pivotti et al. 2011; Tolman and Lewington 2014). Lepidoptera collected at each site were divided by species and by sampling date. For each species at each site, a randomized sub-sample, corresponding to a dry weight of about 100 mg (mean 0.091 g, SD 0.015 g), was set up among the individuals collected at various sampling sessions to obtain a representative sample. Not all the species were found at every site; in total, 36 samples were analysed; according to the number of specimens collected, 15 samples were analysed in single replicate, and 21 were analysed in duplicate. Therefore, depending on the species, the number of individuals per sub-sample corresponded to a range of 4 to 12 (average number of individuals per sample: 4, *L. megera*; 5.4, *P. napi*; 6, *P. rapae*; 11.8, *P. icarus*; 12, *C. pamphilus*), and the average weight of each individual amounted to 18.70, 17.23, 15.15, 8.26, 7.18 mg (d.w.) for *L. megera*, *P. napi*, *P. rapae*, *P. icarus* and *C. pamphilus*, respectively.

### Analytical methods

Before the acid digestion, the samples were subjected to drying in an oven at 105 °C to constant weight for about 16 h. Acid digestion was performed using high-purity acids and reagents (Suprapur® — Supelco), and ultrapure water (18.2 MΩ), 8 mL of HNO_3_ ultrapure 65% and 2 mL of H_2_O_2_ ultrapure 30% were added to each sample, and the mixture was heated up to 170 °C in the MARS 5 microwave system (CEM Corporation) for about 30 min (modified from Nieminen et al. 2001). After cooling to room temperature, ultrapure water was added to the mixture to reach a volume of 25 mL.

Inductively coupled plasma mass spectrometry (ICP-MS) technique was used to measure the metal concentration in mineralized butterfly samples using the triple quadrupole Agilent 8900 ICP-MS-QQQ instrument. The ICP-MS was equipped with an SPS-4 autosampler (Agilent), a cyclonic spray chamber temperature controlled at 2.7 °C, a quartz torch and Ni cones. The acquisition was performed at 1550 W of plasma RF power in Kinetic Energy Discrimination (KED) — general purpose mode, using He as the collision gas (5.5 mL min^−1^). Instrument parameters were optimized for best sensitivity in the whole mass range and minimum oxides (< 1.5%) and double charges (< 1.5%).

Ten trace elements (Al, Cd, Cr, Cu, Fe, Mn, Ni, Pb, Sr and Zn) have been quantitatively determined. To this aim, the instrument was calibrated with standard solutions (ICP multi-element standard solution CertiPUR®, Merck Chemicals and Reagents), obtaining a calibration curve for each metal with the least-squares method (Ordinary Least Squares, OLS). A certified Internal standard mix solution for ICP-MS systems (100 mg L^−1^, 6-Li, Sc, Ge, Rh, In, Tb, Lu, Bi, Agilent Technologies Italia S.p.a.) was also used to correct for changes in ICP-MS operating conditions and sample-specific matrix effects. Limit of detection (LOD) was estimated using the slopes of the calibration lines constructed with certified standard solutions of the examined elements (determination with the conventional least-squares method using the standard deviation of the residuals of the linear regression Sy/x). LOD values are reported in Table S1. Most of the concentrations were above the LOD (only 14 values out of 360 were under the LOD); when the measured values were below it, the LOD value was considered for data analyses. Experimental repeatability was calculated by performing three replicate analyses of two multi-element standard solutions (1 and 10 μg L^−1^). The % RSDs obtained from the repeatability test of the metal solutions were defined as good (0.1–10.0% range).

Details on soil sample collection and sample analysis methods, together with soil elemental concentration data, are reported in the supplementary material of a previous article by Tositti et al. (2020).

### Data analysis

We tested for differences in the ten trace element concentrations among butterfly species collected from all sites using the Kruskal–Wallis test. When the overall *p* value was < 0.05, the intergroup comparisons were conducted by using the Wilcoxon test applying the Bonferroni correction. A correlation analysis between soil and butterfly concentration of trace elements was performed by a bivariate Spearman’s correlation.

All the data from trace element analysis in the butterfly samples were normalized in respect to the maximum concentration observed for that element and species, thus allowing evaluating the spatial trends (range 0–1) independently from the metabolic pattern of each element in the various butterfly species.

Assimilation factors (*AF* = [*metal in the tissues*]/[*metal in the soil*]) were calculated for nine trace elements (Al, Cr, Cu, Fe, Mn, Ni, Pb, Sr and Zn) as the ratio between the trace element concentration in butterflies and the trace element concentration in soil. The AF was not calculated for Cd due to the absence of the corresponding soil data. Soil contamination can influence metal presence in the host plants of butterfly larvae, thus affecting the relative bioaccumulation in adult butterfly tissues. In order to check for trends in the ten elemental concentrations in the five species at the nine sampling sites, the first two axes of a principal component analysis (PCA) were analysed.

Statistical analyses were carried out by means of R Statistical Framework (R Core Team 2021) in the R Studio Environment with the tidyverse, ggpubr and vegan packages (Wickham et al. 2019, Kassambara 2020, Oksanen et al. 2020).

## Results

**Fig. 2:**
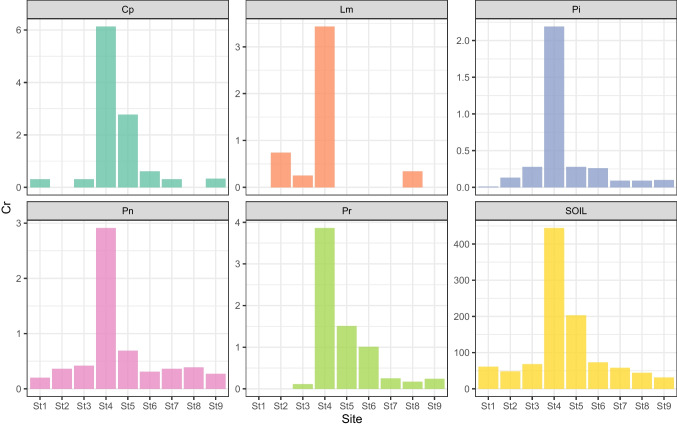
Bar graphs of Cr concentrations (mg kg.^−1^ d.w.) in *Coenonympha pamphilus* (*Cp*), *Lasiommata megera* (*Lm*), *Polyommatus icarus* (*Pi*), *Pieris napi* (*Pn*) and *Pieris rapae* (*Pr*) and in the soil at the nine sampling sites (sites 1–9)

**Fig. 3:**
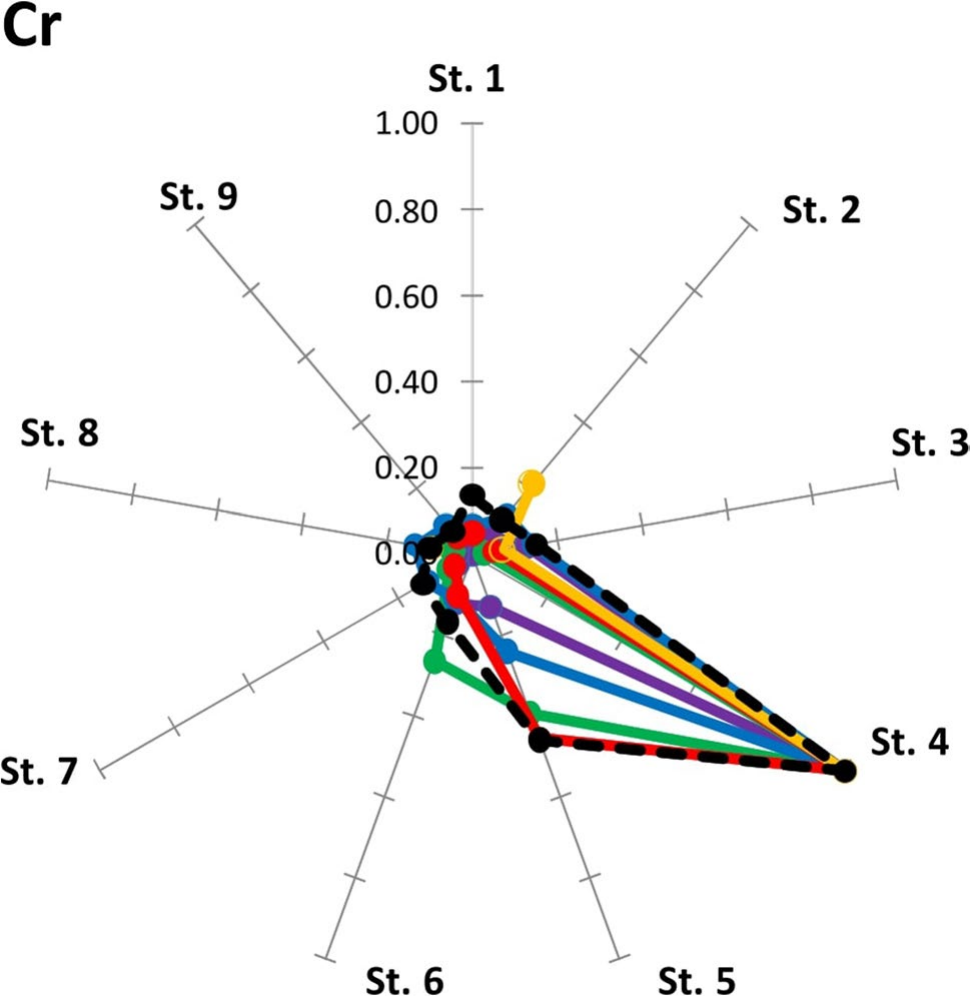
Ratios between the concentrations of Cr with the respective maximum value in the individual species of butterfly (red line, *C. pamphilus*; yellow line, *L. megera*; purple line, *P. icarus*; blue line, *P. napi*; green line, *P. rapae*) and in the soil (black dotted line) at the nine sampling sites (sites 1–9)

The mean concentration values of the ten trace elements in the five butterfly species are reported in Table 1 (the amount of metals for each species is reported in Tab. S1 of the supplementary material). The soil concentration data are taken from Tositti et al. (2020). The bar graphs of the element concentrations in butterflies and soil at the nine sampling sites are reported in Fig. 2 (Cr) and in the supplementary material (Fig. S1-S9); the normalized element concentration values of butterflies and soil at the nine sampling sites are reported in Fig. 3 (Cr) and in the supplementary material (Tab. S2).

**Table 1:** Mean, standard deviation (SD), minimum (Min) and maximum (Max) concentration values of the ten trace metals (Al, Cd, Cr, Cu, Fe, Ni, Mn, Pb, Sr and Zn; mg kg^−1^ d.w.) in the five butterfly species *C. pamphilus*, *L. megera*, *P. icarus*, *P. napi*, *P. rapae*) and in the soil (from Tositti et al. 2020) at the nine sampling sites (sites 1–9)

	Al	Cd	Cr	Cu	Fe	Mn	Ni	Pb	Sr	Zn
*Coenonympha pamphilus*										
Mean	44.98	0.13	1.54	24.63	109.96	14.22	4.47	0.60	3.42	215.29
SD	89.04	0.13	2.21	10.41	67.73	4.58	1.91	0.99	2.35	81.08
Min	0.20	0.03	0.31	15.14	67.46	9.97	2.97	0.00	0.91	161.88
Max	246.22	0.39	6.13	45.78	243.04	21.72	8.44	2.39	8.16	392.45
*Lasiommata megera*										
Mean	6.11	0.03	1.19	19.96	90.47	81.69	4.12	0.98	7.70	259.30
SD	4.84	0.01	1.51	3.71	10.40	27.66	0.83	1.27	1.85	103.42
Min	0.20	0.01	0.25	16.25	75.71	56.96	3.52	0.01	5.05	147.67
Max	10.27	0.04	3.43	23.94	100.11	121.11	5.28	2.82	9.22	369.29
*Pieris napi*										
Mean	11.63	0.30	0.66	28.88	137.02	21.94	1.14	1.69	0.82	151.44
SD	6.78	0.13	0.86	9.04	42.48	6.49	0.95	1.26	0.96	40.49
Min	2.79	0.12	0.20	11.51	51.18	8.91	0.34	0.00	0.01	93.92
Max	22.18	0.54	2.91	35.57	174.59	31.64	3.49	4.07	2.56	201.70
*Pieris rapae*										
Mean	30.03	0.95	1.02	28.01	137.85	14.53	0.45	1.58	0.82	161.11
SD	31.90	0.52	1.36	10.02	68.48	5.96	0.33	1.94	0.65	45.19
Min	1.46	0.09	0.11	13.62	50.47	6.08	0.10	0.00	0.25	100.55
Max	94.80	1.78	3.86	37.42	250.56	22.44	1.02	5.51	1.86	207.26
*Polyommatus icarus*										
Mean	13.68	0.03	0.38	21.55	91.06	13.36	0.95	0.25	2.52	181.05
SD	7.36	0.03	0.69	4.72	22.56	3.05	0.99	0.25	0.91	31.84
Min	7.19	0.01	0.01	14.54	42.20	5.93	0.01	0.00	0.91	101.46
Max	26.37	0.10	2.19	28.10	127.27	16.35	2.74	0.60	3.95	204.39
Soil										
Mean	75,394.44	–	114.44	87.56	17,177.78	1533.33	65.56	88.11	137.11	131.78
SD	33,880.11	–	133.56	32.98	5680.18	964.37	30.47	79.35	107.37	60.66
Min	16,650	–	31	31	7800	700	29	34	52	80
Max	113,000	–	444	140	30,000	3600	133	296	373	245

### Butterflies: trace elements concentration level

#### Coenonympha pamphilus

This butterfly species was not found at sites 2 and 8. A total of 84 specimens were found at the remaining seven sites. In the three sites closer to the steel plant (sites 4, 5 and 6), this species, which is characterized by the lowest mobility among the five species collected, revealed the highest concentrations of all the trace elements (except for Cd) with Cr, Fe and Al peaking at site 4 and Pb, Ni, Cu, Zn and Mn at site 5, respectively. Among all the studied species, *C. pamphilus* showed the highest concentrations of five metals at sites 4 and 5, in particular of Cr with 6.13 mg kg^−1^ (site 4), Ni with 8.44 mg kg^−1^ (site 5), Cu with 45.78 mg kg^−1^ (site 5), Zn with 392.45 mg kg^−1^ (site 5) and Al with 246.22 mg kg^−1^ (site 4).

#### Lasiommata megera

The butterfly was not found at sites 1, 5, 6, 7 and 9. A total of 16 specimens were found at the remaining four sites. This species, characterized by a poor mobility, showed the highest concentrations of Mn, with 121.11 mg kg^−1^ (site 2), and Sr, with 9.22 mg kg^−1^ (site 8). The highest concentrations of Cr and Zn in *L. megera* were observed at site 4, close to the steel plant, where these elements are also very abundant in soils (Tositti et al. 2020).

#### Polyommatus icarus

The species was found at all nine sites for a total of 212 specimens. In the three sites close to the steel plant, the highest concentrations in *P. icarus*, characterized by a moderate dispersal ability, were observed for Cr at site 4, for Cu and Mn at site 5 and for Al at site 6. In comparison with the other observed species, *P. icarus* never showed the maximum concentrations for any metal.

#### Pieris napi

The butterfly was found at all nine sites for a total of 86 specimens. In the three sites closest to the steel plant, this species, characterized by a moderate dispersal ability, showed the highest concentrations of Cr, Cu, Zn and Fe at site 4. In comparison with the other observed species, it never showed the maximum concentrations for any metal.

#### *Pieris rapae*

The butterfly was not found at sites 1 and 2. A total of 72 specimens were found at the remaining seven sites. In the three sites closest to the steel plant, the highest concentrations in *P. rapae*, which can disperse at great distances, were observed for Pb, Cr, Ni, Mn, Fe and Al at site 4 and for Cd at site 6. In comparison with the other observed species, *P. rapae* showed the highest concentrations of Pb with 5.51 mg kg^−1^ (site 2), Cd with 1.78 mg kg^−1^ (site 6) and Fe with 250.56 mg kg^−1^ (site 4).

### From soils to butterflies

The trace element concentration level of the soil observed in the study area (Tositti et al. 2020) revealed a significant anthropic impact at the low-altitude sites (sites 4–6: 133–156 m a.s.l.), because of their proximity to residential and industrial areas, as compared to higher altitude sites (sites 1–3: 338–967 m a.s.l.; sites 7–9: 385–956 m a.s.l.). In particular, among the elements detected in the butterflies, Cr and Sr reached the highest concentrations in the soils of sites 4 and 5, closest to the steel plant. On the other side, the major crustal elements, such as Al and Fe, were widely spread along the transect, in agreement with their lithogenic abundance (Cesari et al. 2012). Pb showed localized maxima in the soil due to specific emission sources, in this case at site 2, due to the presence of an amateur shooting range in this area. This heterogeneity is in part reflected in the element concentrations in the butterfly.

The assimilation factor (AF, indicate the contamination, connecting the environmental and biological matrices through the food web) in all the species of butterflies showed average values for metals lower than 1 except Zn. AF calculation was not performed for Cd, because it was not measured in the soil (Tab. S3).

#### Aluminium

No statistical differences were observed in concentration values among the species (Kruskal–Wallis *χ*^2^ = 4.38, *p* = 0.36). *P. icarus*, *P. rapae*, *P. napi*, *C. pamphilus* and *L. megera* showed the maximum concentrations of Al respectively at site 6 (26.37 mg kg^−1^), at site 4 (94.80 mg kg^−1^), at site 3 (22.18 mg kg^−1^), at site 4 (246.22 mg kg^−1^) and at site 8 (10.27 mg kg^−1^). Al showed the maximum concentration value in the soil at site 8 (1.13 * 10^5^ mg kg^−1^) and is ubiquitous in soils.

#### Cadmium

The Kruskal–Wallis test revealed highly significant differences (Kruskal–Wallis *χ*^2^ = 27.00, *p* < 0.0001) between medians. *P. icarus*, *P. rapae*, *P. napi*, *C. pamphilus* and *L. megera* showed the maximum concentrations of Cd respectively at site 9 (0.10 mg kg^−1^), at site 6 (1.78 mg kg^−1^), at site 7 (0.54 mg kg^−1^), at site 9 (0.39 mg kg^−1^) and at site 2 (0.04 mg kg^−1^). Therefore, diffuse contamination was observed in the study area. Unfortunately, Cd data was not available for the corresponding soil samples.

#### Chromium

The Kruskal–Wallis test revealed significant differences (Kruskal–Wallis *χ*^2^ = 9.50, *p* < 0.049) with noteworthy differences between *P. icarus* versus *C. pamphilus* and *P. rapae* (Wilcoxon test — *p* < 0.05). All five butterfly species, *P. icarus*, *P. rapae*, *P. napi*, *C. pamphilus* and *L. megera*, exhibited the maximum levels of chromium concentration at site 4, with values of 2.19, 3.86, 2.91, 6.13 and 3.43 mg kg^−1^, respectively. These data are in excellent agreement with the data of metals in soil, which showed, precisely at site 4, a particularly high concentration of Cr (444 mg kg^−1^).

#### Copper

No significant differences were found among the species (Kruskal–Wallis *χ*^2^ = 5.21, *p* = 0.266). *P. icarus*, *P. rapae*, *P. napi*, *C. pamphilus* and *L. megera* showed the maximum concentrations of copper respectively at site 5 (28.10 mg kg^−1^), at site 7 (37.42 mg kg^−1^), at site 7 (35.57 mg kg^−1^), at site 5 (45.78 mg kg^−1^) and at site 2 (5.28 mg kg^−1^). The maximum concentration in the soil was observed at site 3 (140.00 mg kg^−1^).

#### Iron

No significant difference was found among the species (Kruskal–Wallis *χ*^2^ = 5.16, *p* = 0.271). *P. icarus*, *P. rapae*, *P. napi*, *C. pamphilus* and *L. megera* showed the maximum concentrations of Fe respectively at site 5 (127.27 mg kg^−1^), at site 4 (250.56 mg kg^−1^), at site 4 (174.59 mg kg^−1^), at site 4 (243.24 mg kg^−1^) and at site 8 (100.11 mg kg^−1^).

In the study area, the butterfly contamination tended to be higher at sites 4 and 5; however, surprisingly, in the soil of these sites, the maximum levels of this metal were not recorded; indeed, Fe showed the maximum concentrations in the soil at site 1 (3.0 * 10^4^ mg kg^−1^).

#### Manganese

The Kruskal–Wallis test showed highly significant differences (Kruskal–Wallis *χ*^2^ = 18.70, *p* < 0.001) with marked different medians between *L. megera* versus *P. napi* (Wilcoxon test — *p* < 0.01), and of both these species versus the other species (Wilcoxon test — *p* < 0.05). *P. icarus*, *P. rapae*, *P. napi*, *C. pamphilus* and *L. megera* showed the maximum manganese concentrations, respectively at site 5 (16.35 mg kg^−1^), at site 4 (22.44 mg kg^−1^), at site 9 (31.64 mg kg^−1^), at site 5 (21.72 mg kg^−1^) and at site 2 (121.11 mg kg^−1^). Mn showed in the soil the maximum concentration at site 1 (3.6 * 10^3^ mg kg^−1^).

#### Nickel

The Kruskal–Wallis test revealed highly significant differences (Kruskal–Wallis *χ*^2^ = 23.58, *p* < 0.0001) between the medians of *C. pamphilus* and *L. megera* showing significantly higher concentrations than the other species (Wilcoxon test — *p* < 0.05). *P. icarus*, *P. rapae*, *P. napi*, *C. pamphilus* and *L. megera* showed the maximum concentrations of Ni respectively at site 7 (2.74 mg kg^−1^), at site 4 (1.02 mg kg^−1^), at site 9 (3.49 mg kg^−1^), at site 5 (8.44 mg kg^−1^) and at site 2 (5.28 mg kg^−1^). Ni showed in the soil the maximum concentration at site 1 (133.00 mg kg^−1^).

#### Lead

No significant differences were found among the species for this element (Kruskal–Wallis *χ*^2^ = 9.47, *p* = 0.051). All the butterflies found at site 2, *P. icarus*, *P. napi* and *L. megera*, showed the maximum concentration levels of lead at this site, respectively, 0.60, 4.07 and 2.82 mg kg^−1^. These data are in excellent agreement with lead occurrence in soil, which showed a peculiarly high content of Pb (296.00 mg kg^−1^) at this site. Butterflies not detected at site 2, *P. rapae* and *C. pamphilus*, showed the maximum Pb concentration at site 4 (5.51 mg kg^−1^) and at site 5 (2.39 mg kg^−1^), respectively.

#### Strontium

The Kruskal–Wallis test indicated highly significant differences (Kruskal–Wallis *χ*^2^ = 22.72, *p* < 0.0001) among the species, with the exception between *C. pamphilus* versus *P. icarus* and *P. napi* versus *P. rapae* (Wilcoxon test — *p* > 0.05). *L. megera*, *C. pamphilus*, *P. icarus*, *P. napi* and *P. rapae* showed the maximum concentrations of Sr respectively at site 8 (9.22 mg kg^−1^), at site 5 (8.16 mg kg^−1^), at site 3 (3.95 mg kg^−1^), at site 9 (2.56 mg kg^−1^) and at site 7 (1.86 mg kg^−1^). For strontium in the study area, there was a tendency of a less localized contamination. Sr showed in the soil the maximum concentration at site 5 (373 mg kg^−1^).

#### Zinc

No significant differences were found among the species (Kruskal–Wallis *χ*^2^ = 6.65, *p* = 0.156). *P. icarus*, *P. rapae*, *P. napi*, *C. pamphilus* and *L. megera* showed the maximum concentrations of Zn respectively at site 7 (204.39 mg kg^−1^), at site 7 (207.26 mg kg^−1^), at site 4 (201.70 mg kg^−1^), at site 5 (392.45 mg kg^−1^) and at site 4 (369.29 mg kg^−1^). Zn showed the maximum soil concentration at site 2 (245.00 mg kg^−1^). Only for Zn it was observed that the assimilation factor in all species of butterflies showed average values higher than 1, between 1.3 (*P. napi*) and 2.0 (*C. pamphilus*).

The correlation between trace metal concentrations in soil and in butterfly species (except for Cd, which was not measured in the soil) (Fig. S10-S18) showed a statistically significant positive correlation between Al and *P. napi* (Fig. S10), between Cr and *P. icarus* (Fig. S11), and between Sr and *C. pamphilus* (Fig. S17). In addition, concerning Cr, we can also observe a clear positive correlation with *C. pamphilus* and *P. rapae*, which is not statistically significant probably because both species were not found in two sites (sites 2 and 8; sites 1 and 2, respectively).

**Fig. 4:**
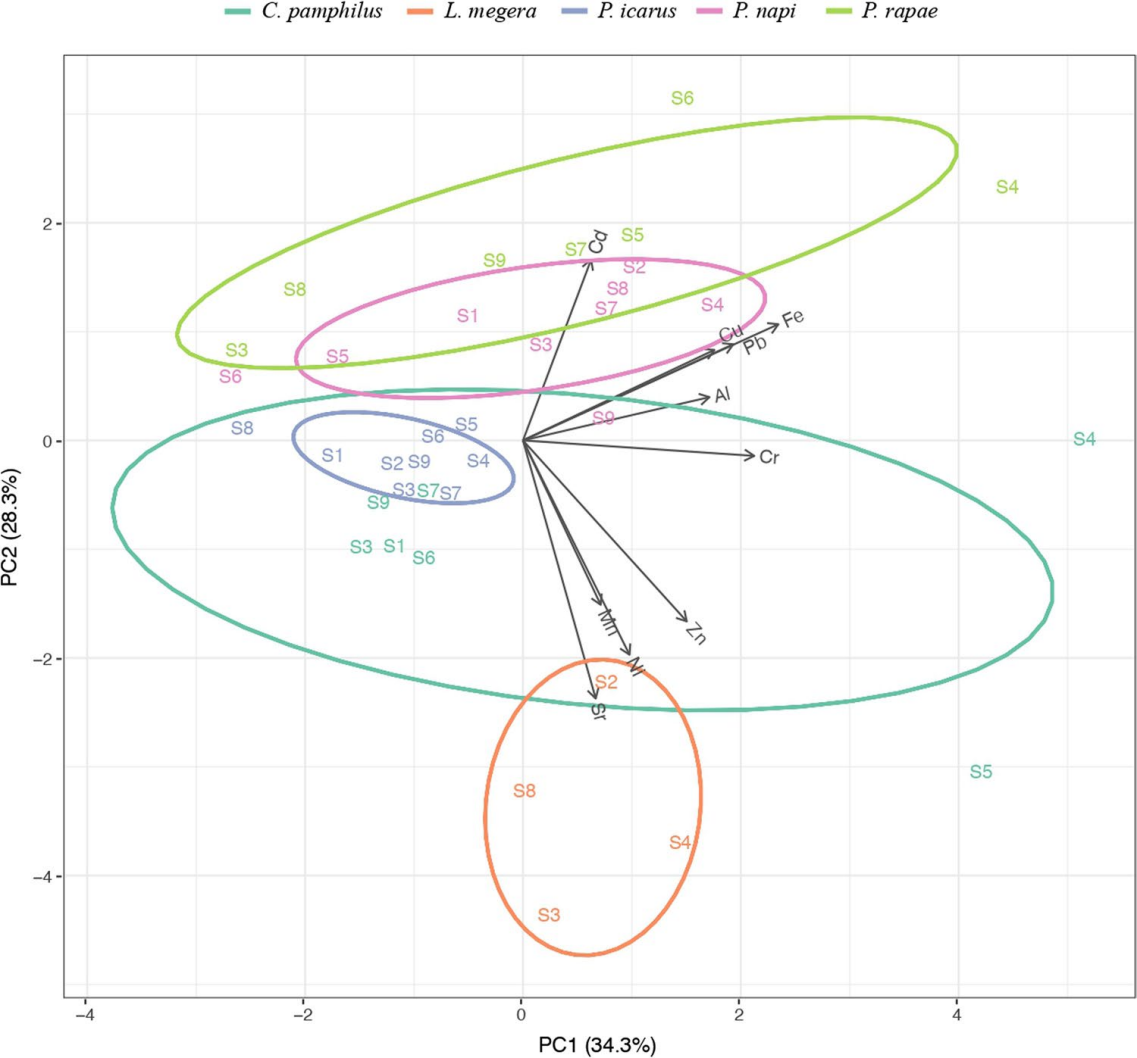
Principal component analysis (PCA) (first two axes, 62.6% of the variance) of the element accumulations (Al, Cd, Cr, Cu, Fe, Ni, Mn, Pb, Sr and Zn) in the five butterfly species (*C. pamphilus*, *L. megera*, *P. icarus*, *P. napi*, *P. rapae*) at the nine sampling sites (sites 1–9)

Principal component analysis (Fig. 4) of the 10 trace metals in the 5 butterfly species explained 62.6% of the variance in the two main axes overall. PC1 axis (34.3%) showed a positive spatial association characterized by the metals Cu, Fe, Pb, Al, Cr and Zn in the sites closer to the industrial area (sites 4–6), such as site 4 for all the five butterfly species; site 5 for *P. rapae*, *P. icarus*, and *C. pamphilus* and site 6 for *P. rapae* and *P. icarus*. A negative spatial association between metals was observed between site 3 and *P. rapae* and *C. pamphilus*; between site 8 and *P. icarus*, *P. rapae*, and *L. megera* and between site 9 and *C. pamphilus*. PC2 axis (28.3%) showed an association between butterflies and metals characterized by a positive relationship between *P. rapae* and *P. napi* and Cd and a negative relationship between *L. megera* with Sr, Ni and Mn.

## Discussion

In terrestrial ecosystems, the Western honey bee (*Apis mellifera* Linnaeus, 1758) plays a major role in studies concerning the bioindication of environmental pollution. Bees, used in apiculture, are reliable bioindicators of environmental contamination, showing clear patterns of contaminant accumulation in their tissues and in beehive products (Devillers and Pham-Delègue 2002; van der Steen et al. 2016; Zhou et al. 2018; Goretti et al. 2020b, 2023). On the other hand, aquatic insects, as indicator species (sensu Beeby 2001), are widely used in freshwater biomonitoring of human impacts, providing helpful information on water and sediment quality through the correlation of their relative occurrence, diversity and abundance to the environmental contamination level (Pallottini et al. 2017b; Goretti et al. 2020a; Arnold et al. 2021).

The present study fits in these contexts in an innovative way, aiming to test the use of different species of butterflies to evaluate the contamination by trace metals along a transect at increasing distances from an urban-industrial area.

The highest levels of metal contamination detected in the Lepidoptera of the Terni basin were comparable to other studies examining adult Lepidoptera at industrial sites (Van San and Spitzer 1993; Bagatto & Shorthouse 1996; Azam et al. 2015; Lin et al. 2019). In particular, the maximum concentrations (mg kg^−1^ d.w.) in the present study were detected for Al (246.22), Cr (6.13), Cu (45.78), Ni (8.44) and Zn (392.45) in *C. pamphilus*; Mn (121.11) and Sr (9.22) in *L. megera* and Cd (1.78), Fe (250.56) and Pb (5.51) in *P. rapae* (Table 2).

**Table 2:** Maximum trace element concentrations in butterfly species from literature studies (mg kg^−1^ d.w.)

Study	Taxon	Country	Sites	Al	Cd	Cr	Cu	Fe	Mn	Ni	Pb	Sr	Zn
**Current study**	5 species	Italy	Relatively pristine spots and industrial areas	246	1.78	6.13	45.8	251	121	8.44	5.51	9.22	392
van San and Spitzer 1993	*Opheroptera brumata* (Linnaeus, 1758)	Czech Republic	Sites with limited anthropogenic impact	–	1	0.6	21.9	161.1	12.1	4.1	3.1	–	338.6
Bagatto and Shorthouse 1996	*Lymantria dispar* (Linnaeus, 1758)	Canada	Areas affected by the pollution from smelters	–	–	–	25–45^a^	–	–	3–8^a^	–	–	–
Azam et al. 2015	*Danaus chrysippus* (Linnaeus, 1758)	Pakistan	Different sites near industrial areas	–	0.45	9	165	–	–	8	–	–	125
Lin et al. 2019	*Spodoptera litura* Fabricius, 1775	China	Sites with intensive tobacco cultivation	392.08	–	9.56	–	641	76.28	–	–	41.49	247.89

^a^First value females — second value males

Besides, Bagatto and Shorthouse (1996) did not find any significant difference between sites (polluted and control) in Cu and Ni concentrations; Azam et al. (2015) found significantly different concentrations for Cd, Cr and Cu among sites with different impact, but no differences for Ni and Zn; Lin et al. (2019) found that Fe and Zn concentrations in butterflies were positively correlated with soil concentrations; a negative correlation was found for Sr, while no correlation was found for Al, Mn and Cr. In addition, Zhou et al. (2015a) analysed lead accumulation in *Bombyx mori* (Linnaeus, 1758) and its translocation in the soil–mulberry–silkworm food chain. Lead concentrations in adults Lepidoptera showed the same trend of the lead content in the leaves of the plants of which the larvae fed and consequently in the soil (in the laboratory, the soil was enriched with lead at increasing concentrations). However, the lead levels in adult butterflies were much lower than those in the larvae. High concentrations of lead were found instead in the faeces of the larvae and in the pupal envelope.

From the analysis of the trace element concentration in the present study, we observed that the butterfly species examined are to be considered good bioindicators for chromium. In sites where the soil contamination was high for Cr, consequently, there were higher contamination levels in butterfly tissues, while a clear decrease of Cr concentration in butterflies at increasing distances from site 4, both toward site 1 and site 9, is observed (Fig. 2).

In the soil of site 4, Cr showed a concentration about 13 times higher than the crustal background (Upper Continental Crust; Rudnick and Gao 2003) (Tab. S1) due to its proximity to the steel plant. The Cr concentration level was also relatively high at site 5 (about 6 times the crustal background), in turn close to the plant as site 4. The average level of soil concentration in the remaining sites with increasing distance from the industrial plant was instead close to the crustal background (about 1.5 times).

In fact, Cr contamination in the Terni basin is of particular concern in the areas surrounding the steel plant, being this metal linked to the industrial production activity of the plant (Moroni et al. 2012; ARPA Valle d'Aosta et al. 2018). This condition can be well evidenced by Fig. 3, where the ratios between Cr concentrations and their maximum values in each butterfly species and in the soils of Terni basin transect always showed a value of 1 at site 4, the closest site to the AST steel plant.

However, the sensitivity to this metal differed among the different butterfly species (Fig. 2).

From the analysis of the regression lines, confidence intervals (95%) for intercept and slope showed, in absolute value, a decreasing sequence among the species: *C. pamphilus* < *P. rapae* < *P. icarus* < *P. napi* < *L. megera* (Tab. S4; analysis of residuals, Figs. S11a-e), thus highlighting that *C. pamphilus* is the best species for Cr, in terms of accumulation in body tissues. On the other hand, in the case of Pb, despite the high levels of the metal in the butterfly tissues at the heavily contaminated site (site 2), unlike for Cr, the relationship between soil and butterflies are inconsistent for sites with soil concentrations below 100 mg kg^−1^ (sites 1, 3–9).

Lead showed maximum concentrations at site 2, both in the only three butterfly species found (*P. icarus*, *P. napi* and *L. megera*) and in soil. Pb reveals further relative maxima at sites 4 and 5 only for those species of butterflies that were not found at site 2 (*P. rapae* and *C. pamphilus*). Interestingly, in the soil, Pb showed a concentration about 17 times higher than the crustal background (Upper Continental Crust; Rudnick and Gao 2003) at site 2 (Tab. S1), due to the presence in the past years of an amateur shooting range close to this site. This past land use caused the typical contamination signature due to the lead contained in the bullets (Hardison et al. 2004; Laidlaw et al. 2017). However, the Pb concentration level is relatively high (an average of 5.2 times of the crustal background) also in all the other sites, suggesting widespread anthropogenic lead contamination connected to atmospheric source emission with subsequent dispersion in the aerosol phase and successive wet-dry deposition (Hernberg 2000). This phenomenon of widespread Pb contamination in the Terni basin valley could still be due to the past use of leaded gasoline, banned in Italy only in 2001. This condition was also found in other species of animals frequenting areas affected by Pb contamination coming from neighbouring urban areas, such as in the mustelids of central Italy (Goretti et al. 2018).

However, regardless of the particular environmental conditions of sites 2 and 4, we would have expected that the metal concentration in the tissues of the five butterfly species would have shown an increase at the closest sites to the steel plant, in particular at sites 4–6, only about 1–2.4 km far from the production activities of the steel plant AST. The PCA provided a graphic summary of the metal contamination in butterfly species along the transect under study. In particular, PC1 axis showed a positive association among butterfly trace element concentrations (in particular Cr) and the closest sites to the industrial area. Cr showed a strong association to PC1 and no association with PC2, confirming that Cr in butterflies is controlled exclusively by the anthropogenic gradient. The anthropogenic origin of contamination is less evident for Al, Cu, Pb and Fe. On the other hand, not all butterfly species responded to the contamination in the same way when trace element concentrations were not particularly high, in fact, PC2 axis showed a relative trend of sensitivity of butterfly species to various trace elements (i.e. Cd, Sr, Mn, Ni and Zn).

In this regard, the species that emerged as the best in detecting metal contamination was *C. pamphilus*, which always showed the highest concentration values for all the ten metals at sites 4 and 5. In addition, these values were the maximum values of contamination among all butterfly species for Cr and Al at site 4, and for Ni, Cu and Zn at site 5. This species was not found at site 2; therefore, the peculiar lead contamination caused by the firing range at this site could not be detected. *C. pamphilus* is a very common species, ubiquitous and with the lowest mobility among the species examined; its widespread presence is due to the fact that the plants of the family Poaceae, the host plants of its larvae, are very common in all the habitat types. The Poaceae are plants capable of accumulating high amounts of metals in the rhizosphere (Patra et al. 2021), and partially transferring them to the aerial parts (e.g. shoot and leaves; Pedron et al. 2009; Barbafieri et al. 2011), of which larvae of this species feed on, thus determining their transfer into the food chain.

In addition, in *P. rapae*, the highest concentration values among all butterfly species were detected for Pb and Fe at site 4, and for Cd at site 6. The Brassicaceae family is the host plants of the larvae of *P. rapae*, and this species frequents numerous types of habitat, and it can be a migratory species. The only metals with concentration peaks in sites far from the steel plant were Mn at site 2 and Sr at site 8 in *L. megera*. This species has the Poaceae family as the host plants of its larvae.

Finally, some species of butterflies may have migration trends, so the tissue contamination of their adult stages may not be related to the environmental conditions of the survey areas but coming from different locations. In this regard, among the five species of butterflies examined, only *P. rapae* can have migratory tendencies. However, this behavioural characteristic does not seem to be fully expressed for this species in the study area, in particular for Cr, which showed concentrations in its tissues closely connected with the contamination of the Terni basin transect.

## Conclusions

The metal concentration analysis showed that the butterfly species examined can be considered good bioindicators of environmental metal contamination in sites where the contamination conditions are high, as for Cr in the current study. However, a specific ability to detect contamination among the different species was observed, probably due to their peculiar dispersal abilities, diets and metabolic pathways. Indeed, the different levels of metal assimilation of the larvae host plants determine fairly diversified access to the various metals along the food web, influenced mainly by the sedentary tendency of the species in contaminated sites. Further studies aimed to increase the knowledge on metal pathways influencing butterfly species contamination, also focusing on their diet and on their mobility, will help to define better which butterfly species could be more suitable to be used as bioindicator.

Therefore, assessing the metal concentration levels in some Lepidoptera species might prove as an efficient index and a convenient monitoring tool of environmental quality, including predictability for human health, capable of detecting the extent and trend of metal pollution spatially and overtime in critical areas subject to heavy loads of contaminants.

## Supplementary Information

Below is the link to the electronic supplementary material.

Supplementary file1 (DOCX 3528 KB)

## Data Availability

The datasets used in the study are available from the corresponding author on reasonable request.
