# Supplementary material for: Butterflies as bioindicators of metal contamination
Source: Environ Sci Pollut Res Int. 2023 Aug 8;30(42):95606–20. doi: 10.1007/s11356-023-28930-x (PMC10482766; doi:10.1007/s11356-023-28930-x)

**SUPPLEMENTARY MATERIAL**

*Bio-ecology of the investigated butterfly species*

All the biological and ecological features of the five species reported below refer to the following scientific literature: (IBC 2009; Pivotti et al. 2011; Tolman and Lewington 2014; Mazzei et al. 2021). All the species are very common and ubiquitous in Umbria.

*Coenonympha pamphilus (Linnaeus, 1758), common name “Small Heath”.* Its life cycle is bi-trivoltine, and the adult flight period is between April and October. It overwinters as a larva. The average length of the front wing is between 14 and 18 mm. This species is found from the plains to high altitudes with abundant populations, and it shows a poor mobility.

Diet: the Poaceae family represents the host plants of the larvae.

*Lasiommata megera (Linnaeus, 1767), common name “Wall Brown”.* Its life cycle is bi-trivoltine, and the adult flight period is between April and October. It overwinters as a larva. It frequents grassy and rocky slopes and gullies, flowery meadows, wooded clearings with open stony soils, up to over 2000 m a.s.l., showing a poor mobility. The average length of the front wing is between 19 and 25 mm. The species is found at low altitudes, especially on the banks of rivers and in plain meadows; in the mountains, it is found in the meadows adjacent to the woods, with small populations.

Diet: the Poaceae family represents the host plants of the larvae.

*Pieris napi (Linnaeus, 1758), common name “Green-veined White”.* Its life cycle is at least bi-trivoltine, and the adult flight period is between April and September. It overwinters as a pupa. It frequents humid, grassy, and flowery places with a certain shade, edges of woods, hedges, fertile meadows, wooded valleys from the basal level up to 2000 m a.s.l., showing a moderate dispersal ability. The average length of the front wing is between 19 and 27 mm. This species is very abundant and uniformly distributed both in the plains and in the mountains, often linked to wetlands or the edges of the woods.

Diet: the Brassicaceae family represents the host plants of the larvae.

*Pieris rapae (Linnaeus, 1758), common name “Small White”.* Its life cycle is polyvoltine, and the adult flight period is between March and November. It overwinters as a pupa. It frequents numerous types of habitats from the basal level up to over 2000 m a.s.l.; it is a wandering species and can disperse at great distances through migration The average length of the front wing is between 20 and 28 mm. It can constitute very abundant populations, and also colonize urban environments.

Diet: the Brassicaceae family represents the host plants of the larvae.

*Polyommatus icarus (Rottemburg, 1775), common name “Common Blue”.* The larvae of this species are cared for by various ant species. Its life cycle is polyvoltine, and the adult flight period is between March and November. It overwinters as a larva. It frequents numerous habitats, from the basal level up to over 2000 m a.s.l., showing a moderate dispersal ability. The average length of the front wing is between 13 and 17 mm. It is found from the plain to high altitudes; it also frequents agricultural and urban habitats.

Diet: the Fabaceae family represents the host plants of the larvae.

Tab. S1 – Concentrations, mean, standard deviation (SD) and relative standard deviation (RSD) values of the ten trace metals (Al, Cd, Cr, Cu, Fe, Mn, Ni, Pb, Sr and Zn; mg kg^-1^ d.w.) in *Coenonympha pamphilus* (*Cp*)*, Lasiommata megera* (*Lm*), *Polyommatus icarus* (*Pi*), *Pieris napi* (*Pn*) and *Pieris rapae* (*Pr*) and in the soil at the nine sampling sites (site 1-9);

UCC: upper continental crust; LOD: Limit of Detection.

Tab. S2 - Normalized concentration values (ratios between element concentrations and their respective maximum values in each butterfly species) of ten trace elements (Al, Cd, Cr, Cu, Fe, Mn, Ni, Pb, Sr and Zn) in *Coenonympha pamphilus* (*Cp*)*, Lasiommata megera* (*Lm*), *Polyommatus icarus* (*Pi*), *Pieris napi* (*Pn*) and *Pieris rapae* (*Pr*) and in the soil at the nine sampling sites (site 1-9).

Tab. S3 – Assimilation Factors (AF) of nine trace elements (Al, Cr, Cu, Fe, Mn, Ni, Pb, Sr and Zn) between *Coenonympha pamphilus* (*Cp*)*, Lasiommata megera* (*Lm*), *Polyommatus icarus* (*Pi*), *Pieris napi* (*Pn*) and *Pieris rapae* (*Pr*) and in the soil at the nine sampling sites (site 1-9).

Fig. S1 - Bar graphs of Al concentrations (mg kg^-1^ d.w.) *Coenonympha pamphilus* (*Cp*)*, Lasiommata megera* (*Lm*), *Polyommatus icarus* (*Pi*), *Pieris napi* (*Pn*) and *Pieris rapae* (*Pr*) and in the soil at the nine sampling sites (site 1-9).


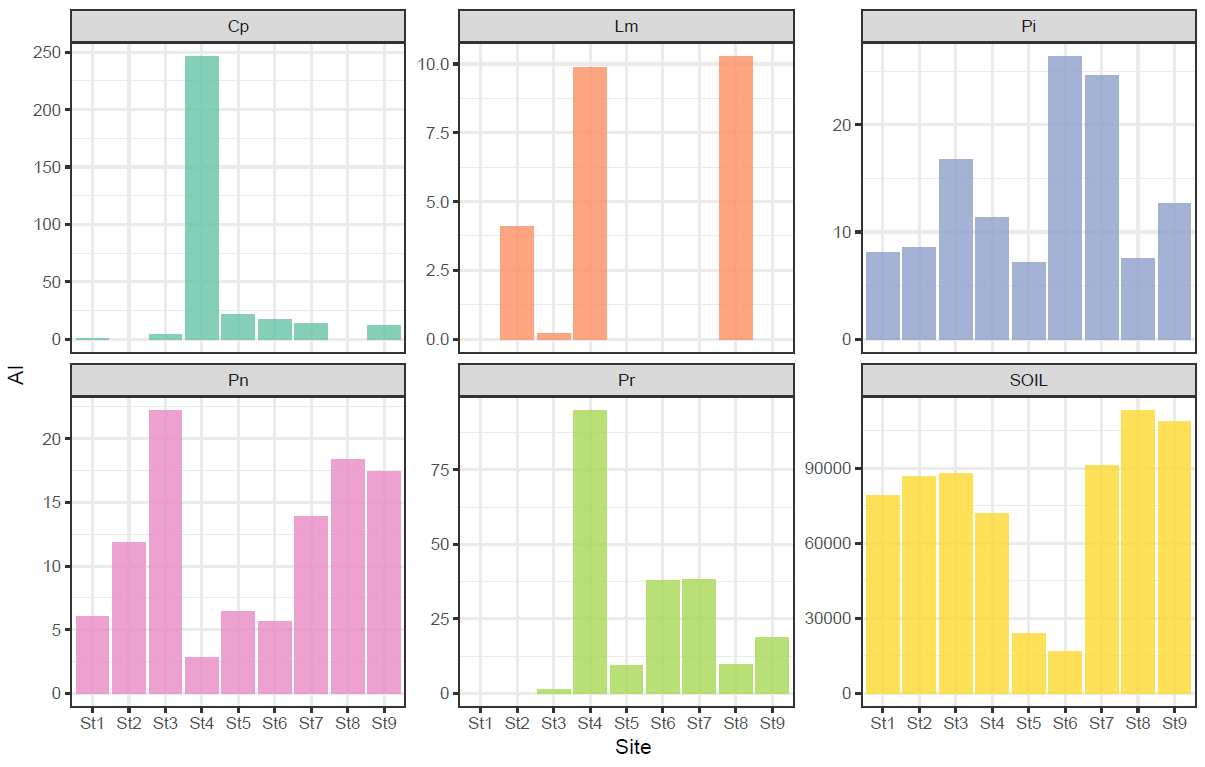


Fig. S2 - Bar graphs of Cd concentrations (mg kg^-1^ d.w.) in *Coenonympha pamphilus* (*Cp*)*, Lasiommata megera* (*Lm*), *Polyommatus icarus* (*Pi*), *Pieris napi* (*Pn*) and *Pieris rapae* (*Pr*) at the nine sampling sites (site 1-9).


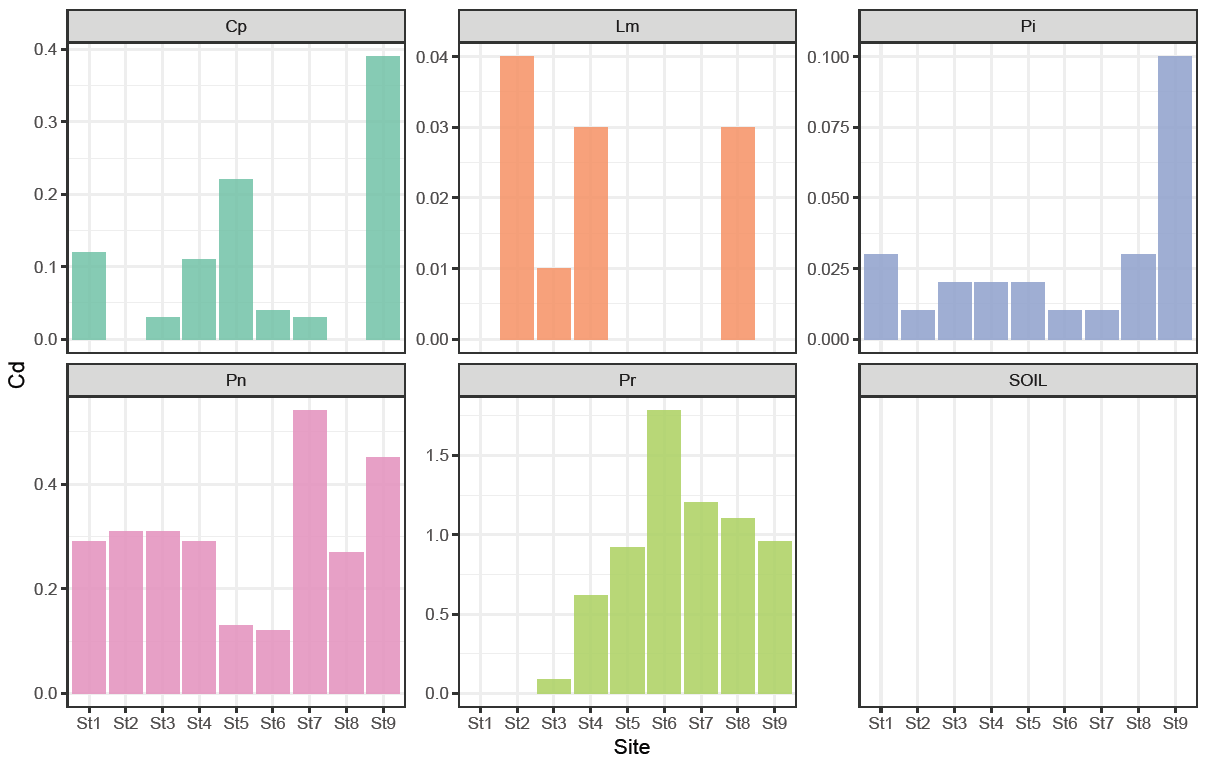


Fig. S3 - Bar graphs of Cu concentrations (mg kg^-1^ d.w.) in *Coenonympha pamphilus* (*Cp*)*, Lasiommata megera* (*Lm*), *Polyommatus icarus* (*Pi*), *Pieris napi* (*Pn*) and *Pieris rapae* (*Pr*) and in the soil at the nine sampling sites (site 1-9).


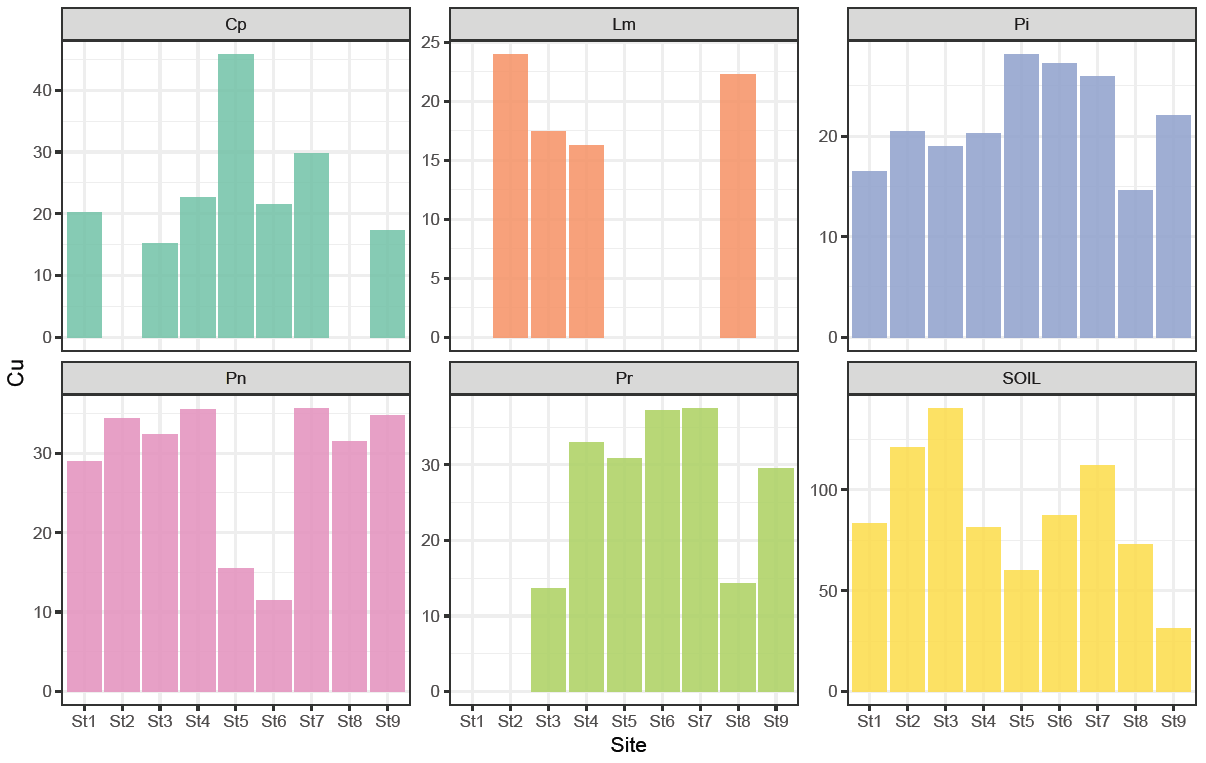


Fig. S4 - Bar graphs of Fe concentrations (mg kg^-1^ d.w.) in *Coenonympha pamphilus* (*Cp*)*, Lasiommata megera* (*Lm*), *Polyommatus icarus* (*Pi*), *Pieris napi* (*Pn*) and *Pieris rapae* (*Pr*) and in the soil at the nine sampling sites (site 1-9).


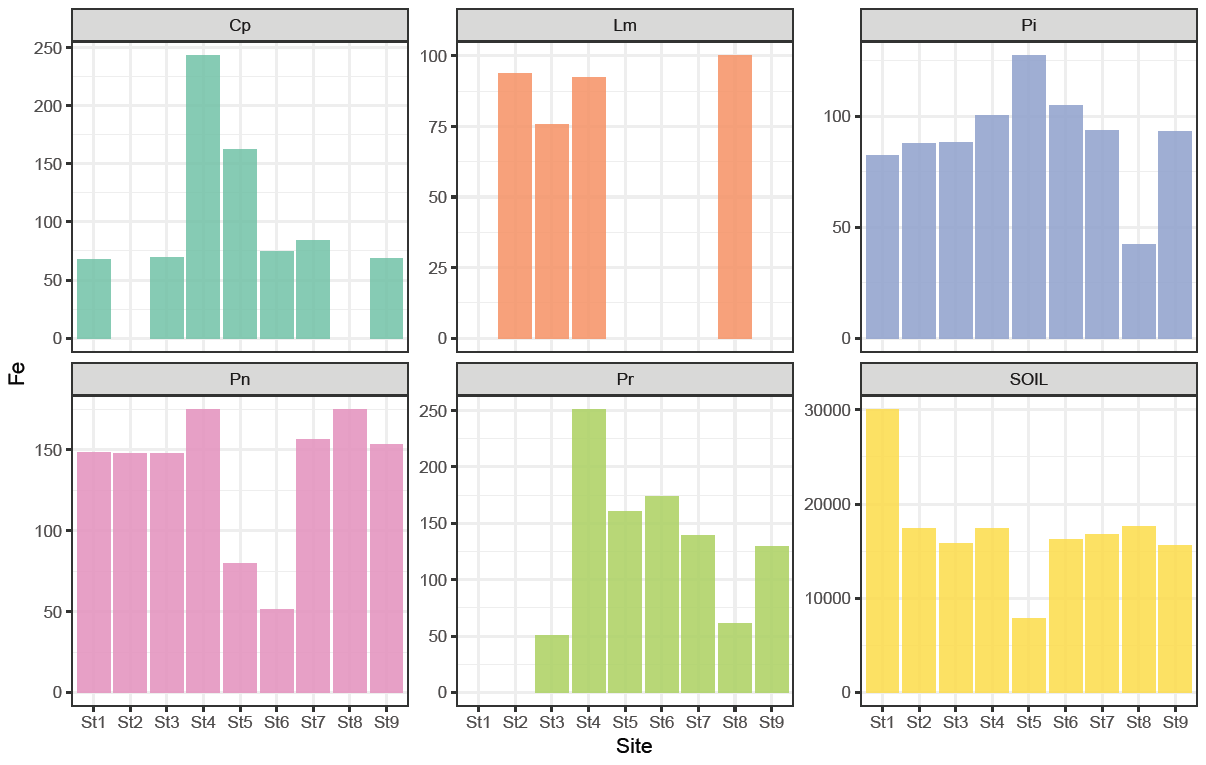


Fig. S5 - Bar graphs of Mn concentrations (mg kg^-1^ d.w.) in *Coenonympha pamphilus* (*Cp*)*, Lasiommata megera* (*Lm*), *Polyommatus icarus* (*Pi*), *Pieris napi* (*Pn*) and *Pieris rapae* (*Pr*) and in the soil at the nine sampling sites (site 1-9).


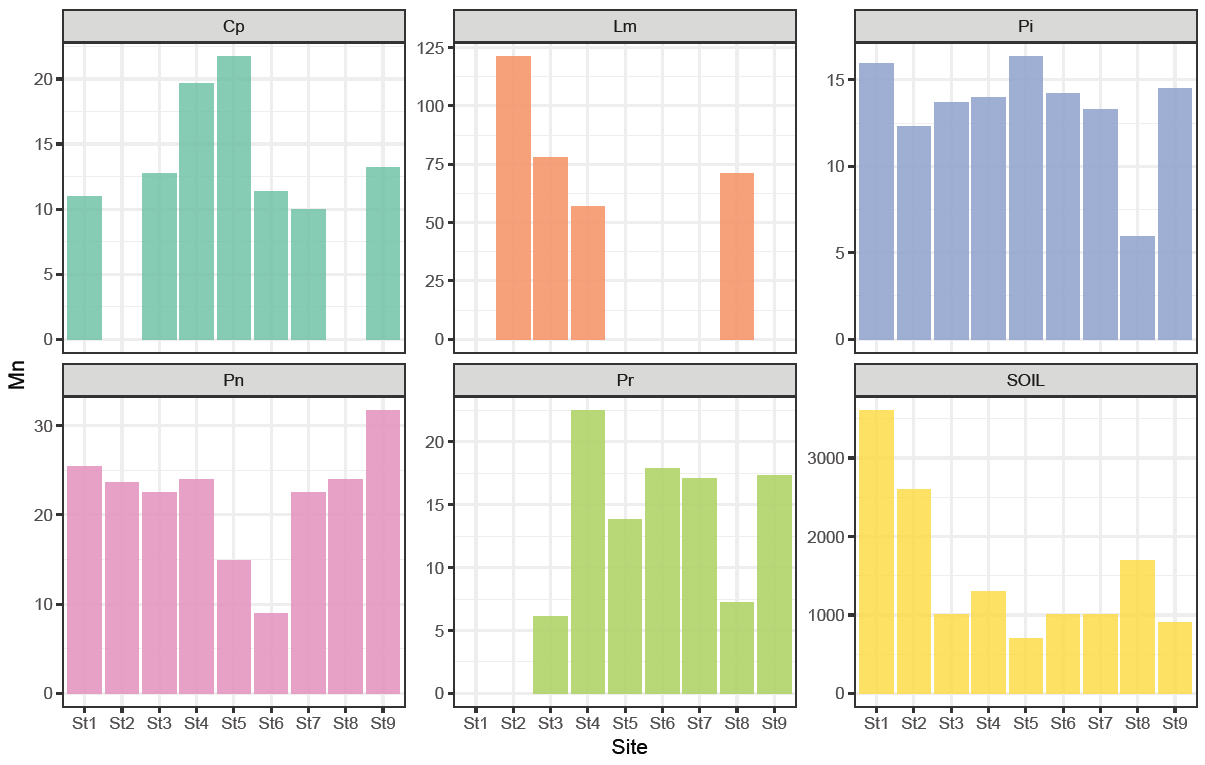


Fig. S6 - Bar graphs of Ni concentrations (mg kg^-1^ d.w.) in *Coenonympha pamphilus* (*Cp*)*, Lasiommata megera* (*Lm*), *Polyommatus icarus* (*Pi*), *Pieris napi* (*Pn*) and *Pieris rapae* (*Pr*) and in the soil at the nine sampling sites (site 1-9).


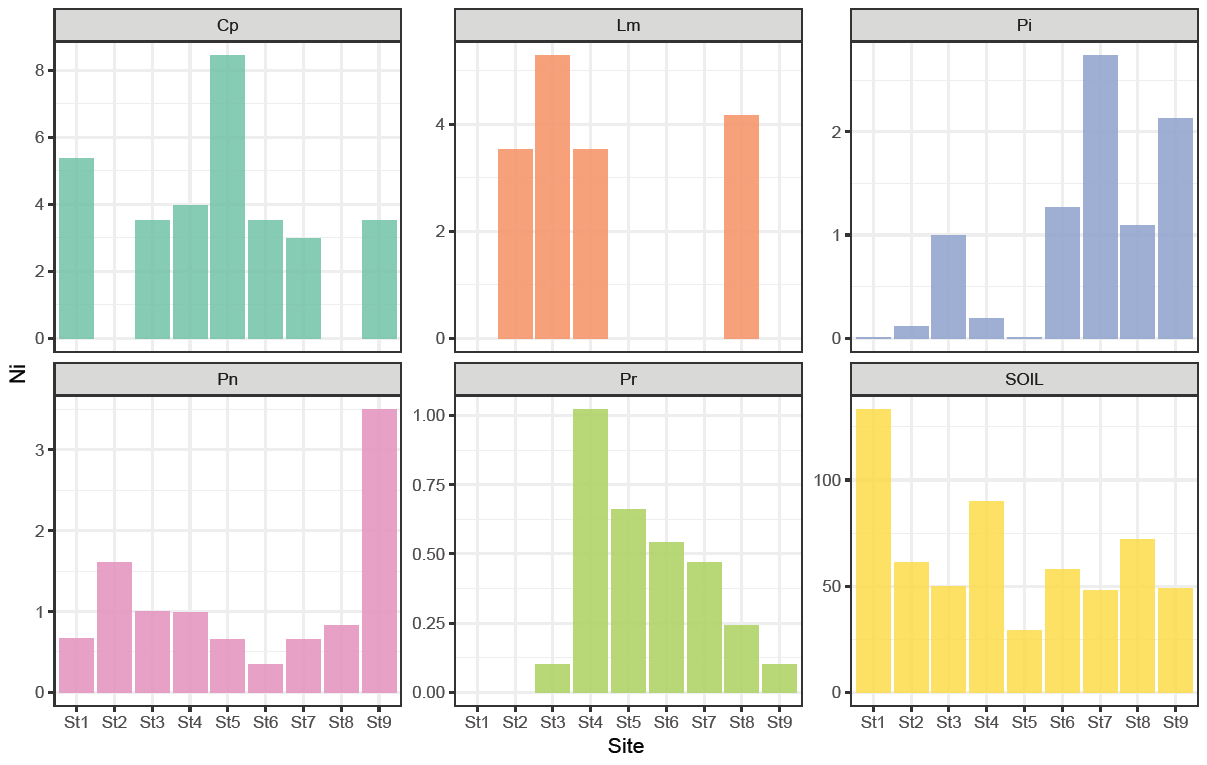


Fig. S7 - Bar graphs of Pb concentrations (mg kg^-1^ d.w.) in *Coenonympha pamphilus* (*Cp*)*, Lasiommata megera* (*Lm*), *Polyommatus icarus* (*Pi*), *Pieris napi* (*Pn*) and *Pieris rapae* (*Pr*) and in the soil at the nine sampling sites (site 1-9).


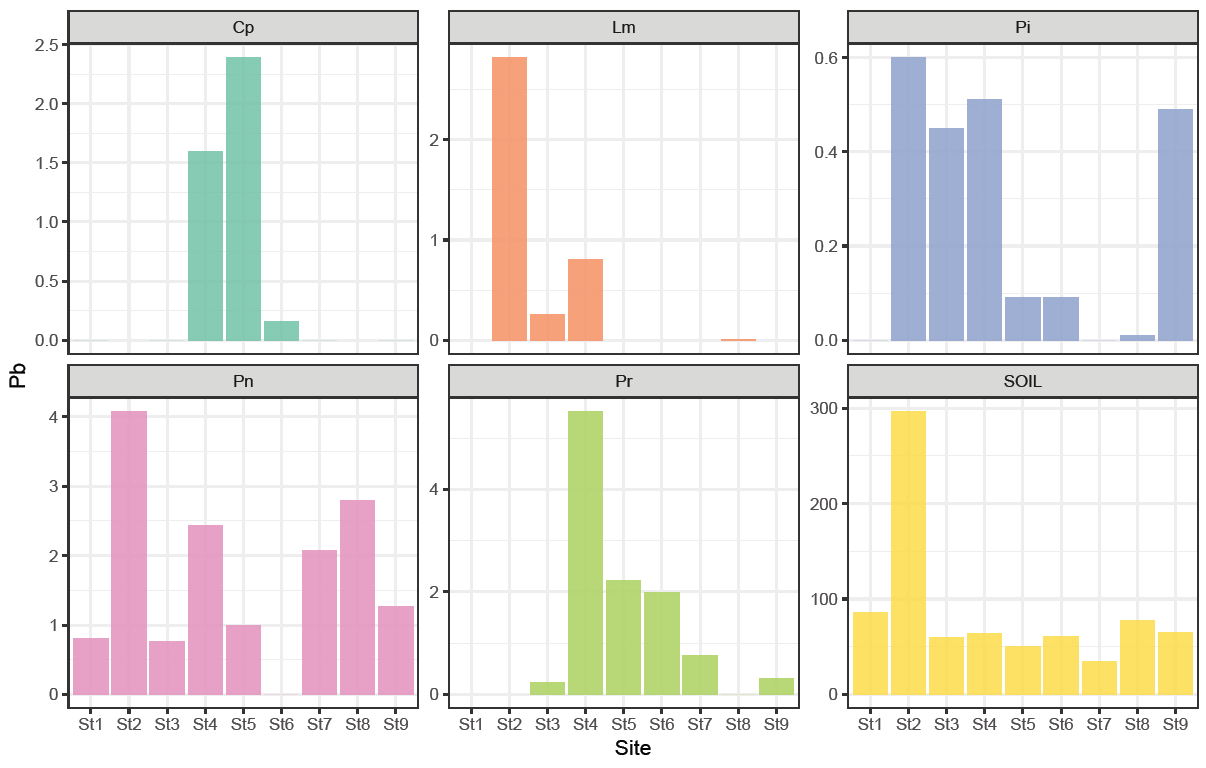


Fig. S8 - Bar graphs of Sr concentrations (mg kg^-1^ d.w.) in *Coenonympha pamphilus* (*Cp*)*, Lasiommata megera* (*Lm*), *Polyommatus icarus* (*Pi*), *Pieris napi* (*Pn*) and *Pieris rapae* (*Pr*) and in the soil at the nine sampling sites (site 1-9).


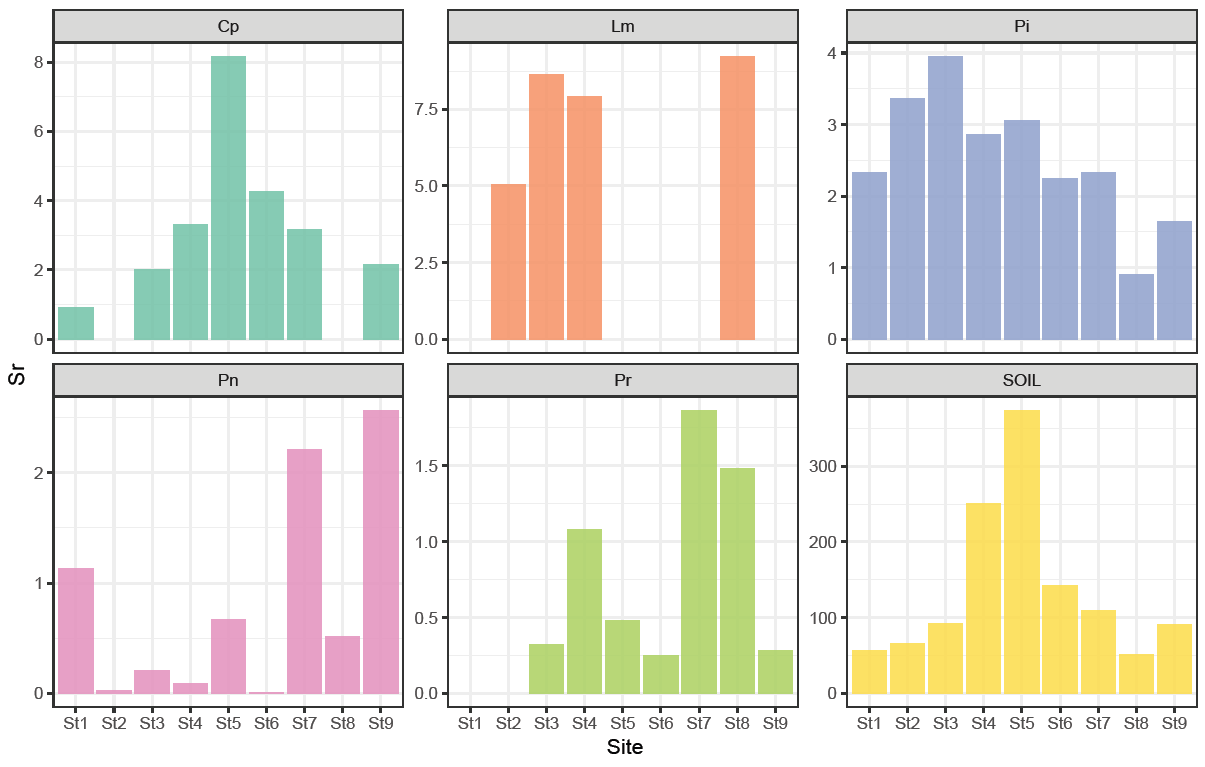


Fig. S9 - Bar graphs of Zn concentrations (mg kg^-1^ d.w.) in *Coenonympha pamphilus* (*Cp*)*, Lasiommata megera* (*Lm*), *Polyommatus icarus* (*Pi*), *Pieris napi* (*Pn*) and *Pieris rapae* (*Pr*) and in the soil at the nine sampling sites (site 1-9).


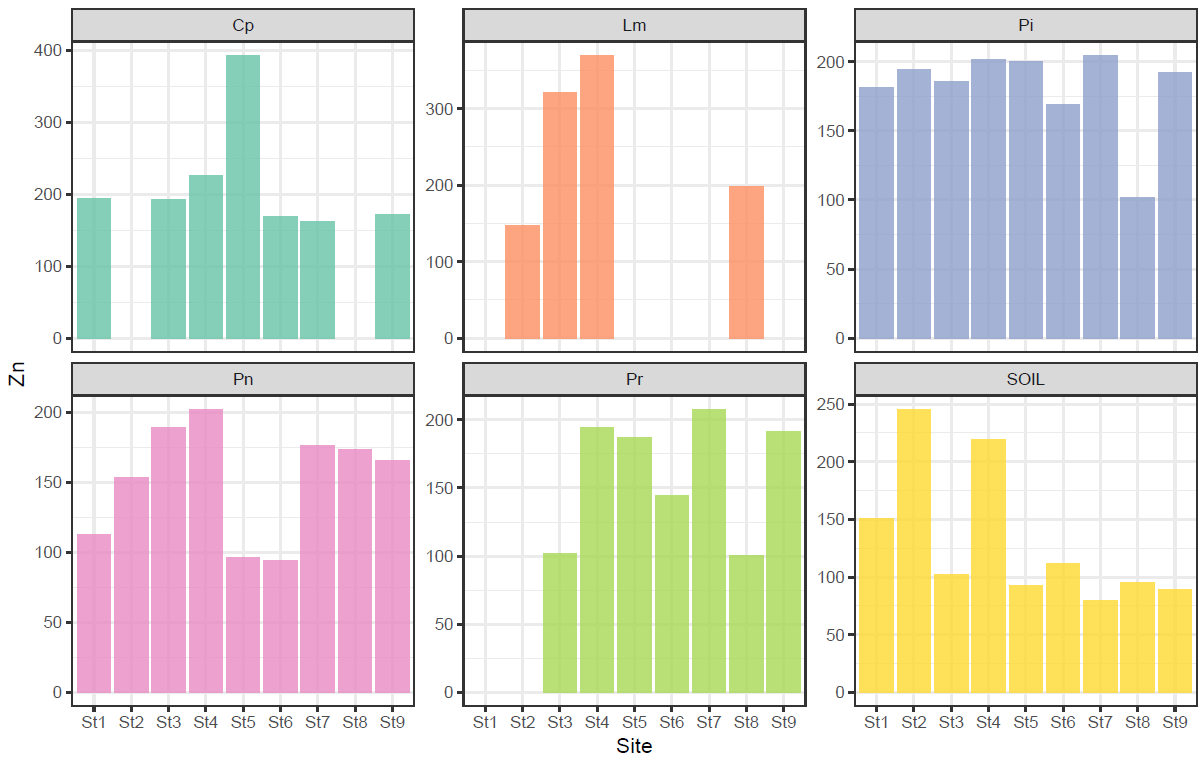


Fig. S10 – Aluminum: bivariate Spearman’s correlation between soil and butterfly concentrations (mg kg^-1^ d.w.) (*Coenonympha pamphilus,* Cp; *Lasiommata megera,* Lm*;* *Polyommatus icarus,* Pi*;* *Pieris napi,* Pn; *Pieris rapae,* Pr).


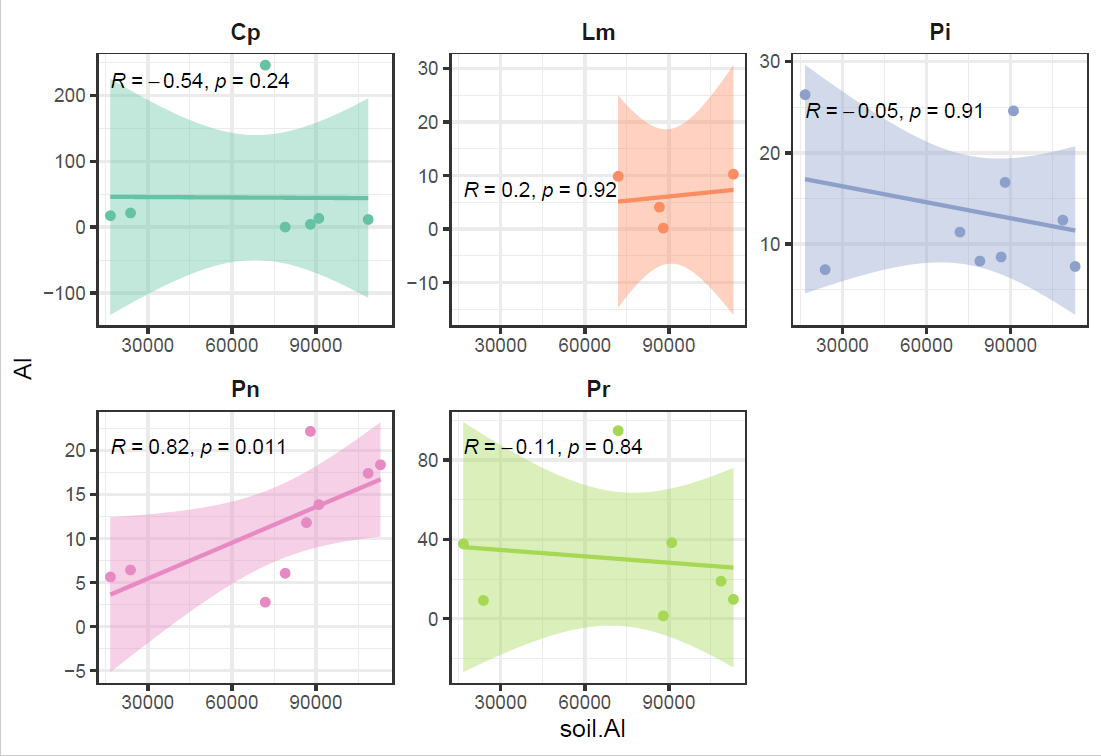


Fig. S11 – Chromium: bivariate Spearman’s correlation between soil and butterfly concentrations (mg kg^-1^ d.w.) (*Coenonympha pamphilus,* Cp; *Lasiommata megera,* Lm*;* *Polyommatus icarus,* Pi*;* *Pieris napi,* Pn; *Pieris rapae,* Pr).


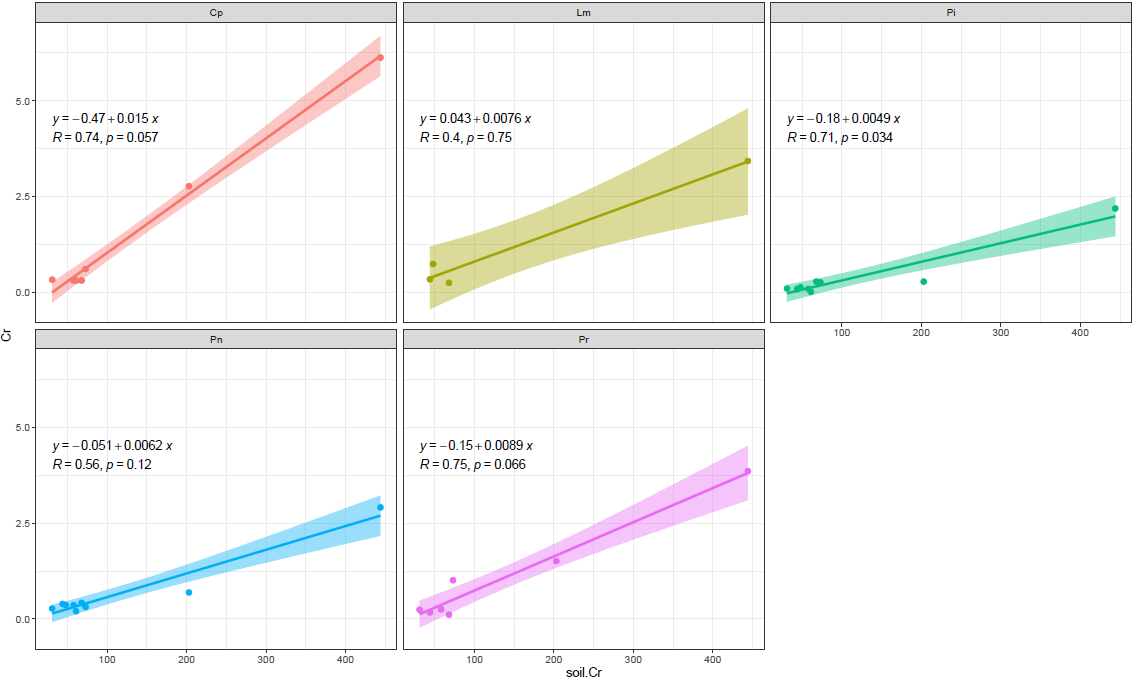


S11a – Residual analysis of *Coenonympha pamphilus*


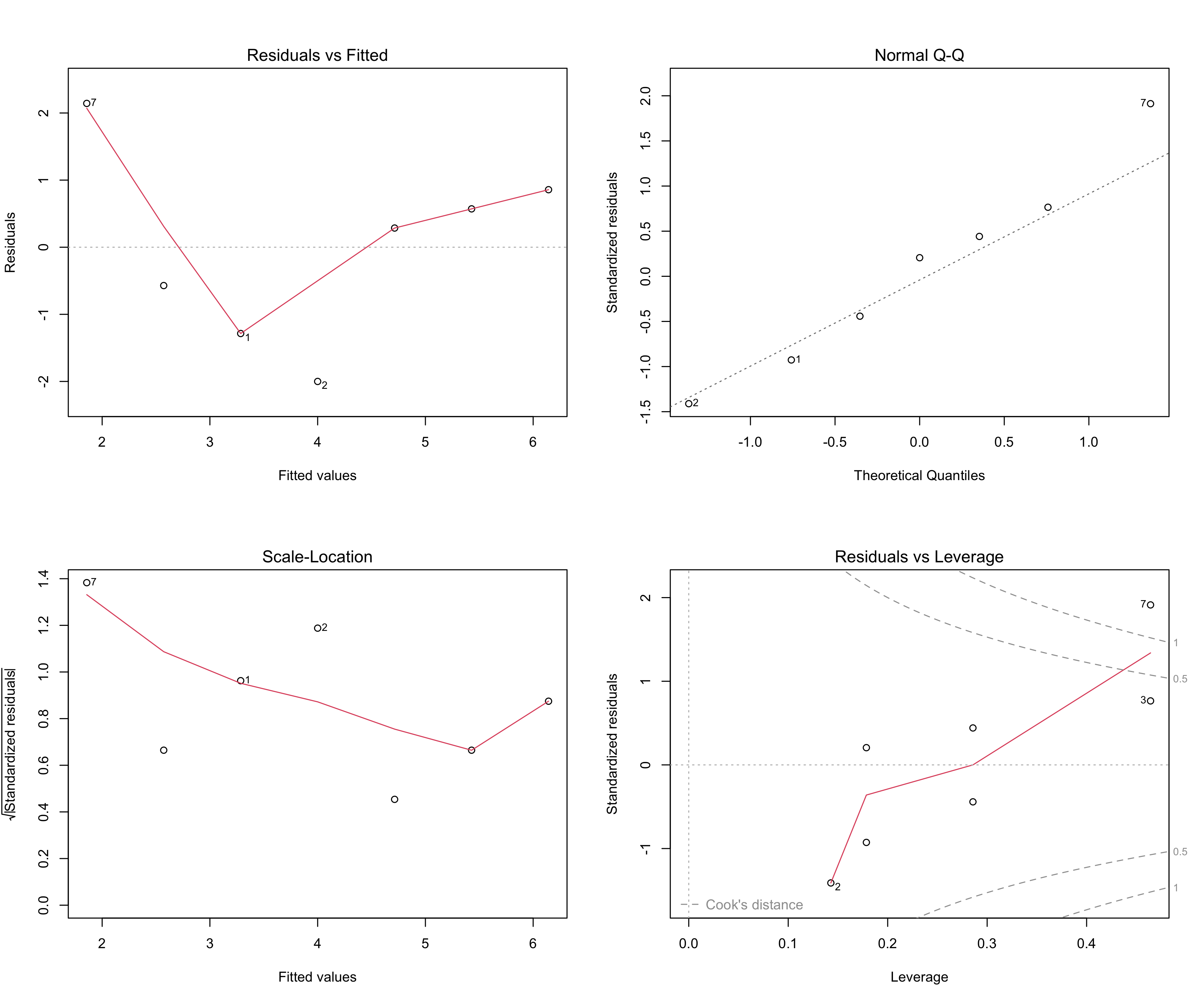


S11b – Residual analysis of *Lasiommata megera*


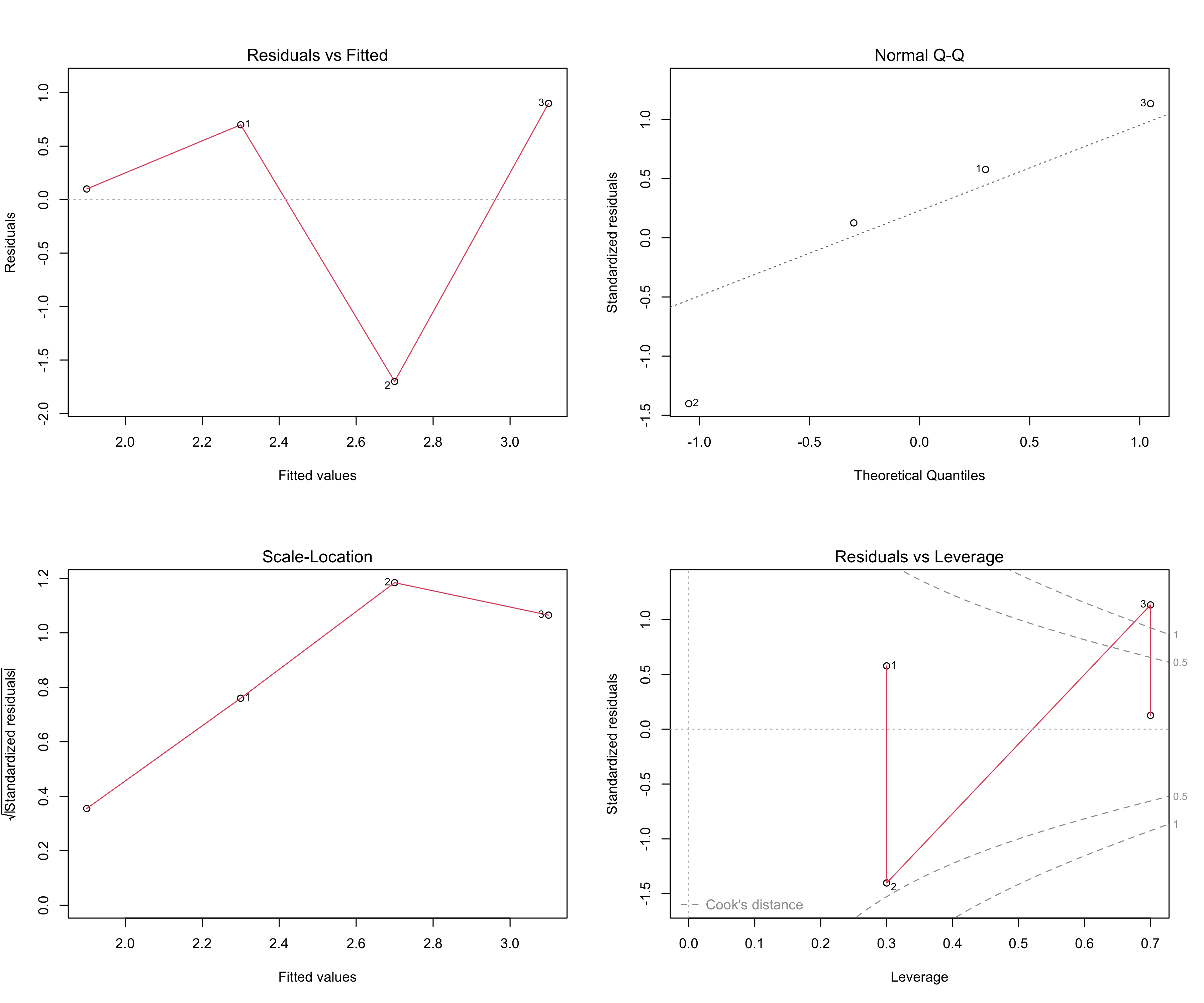


S11c – Residual analysis of *Polyommatus icarus*


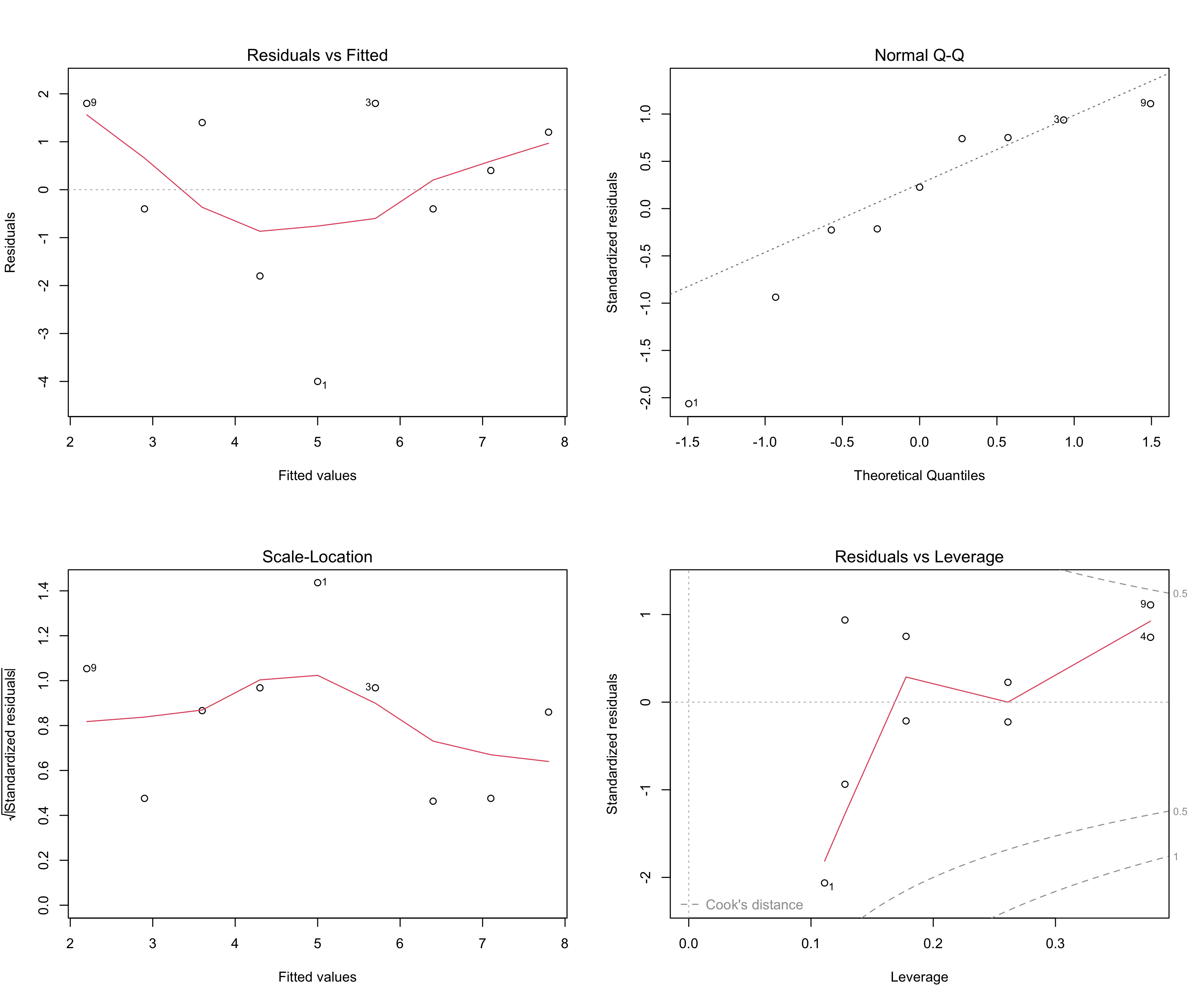


S11d – Residual analysis of *Pieris napi*


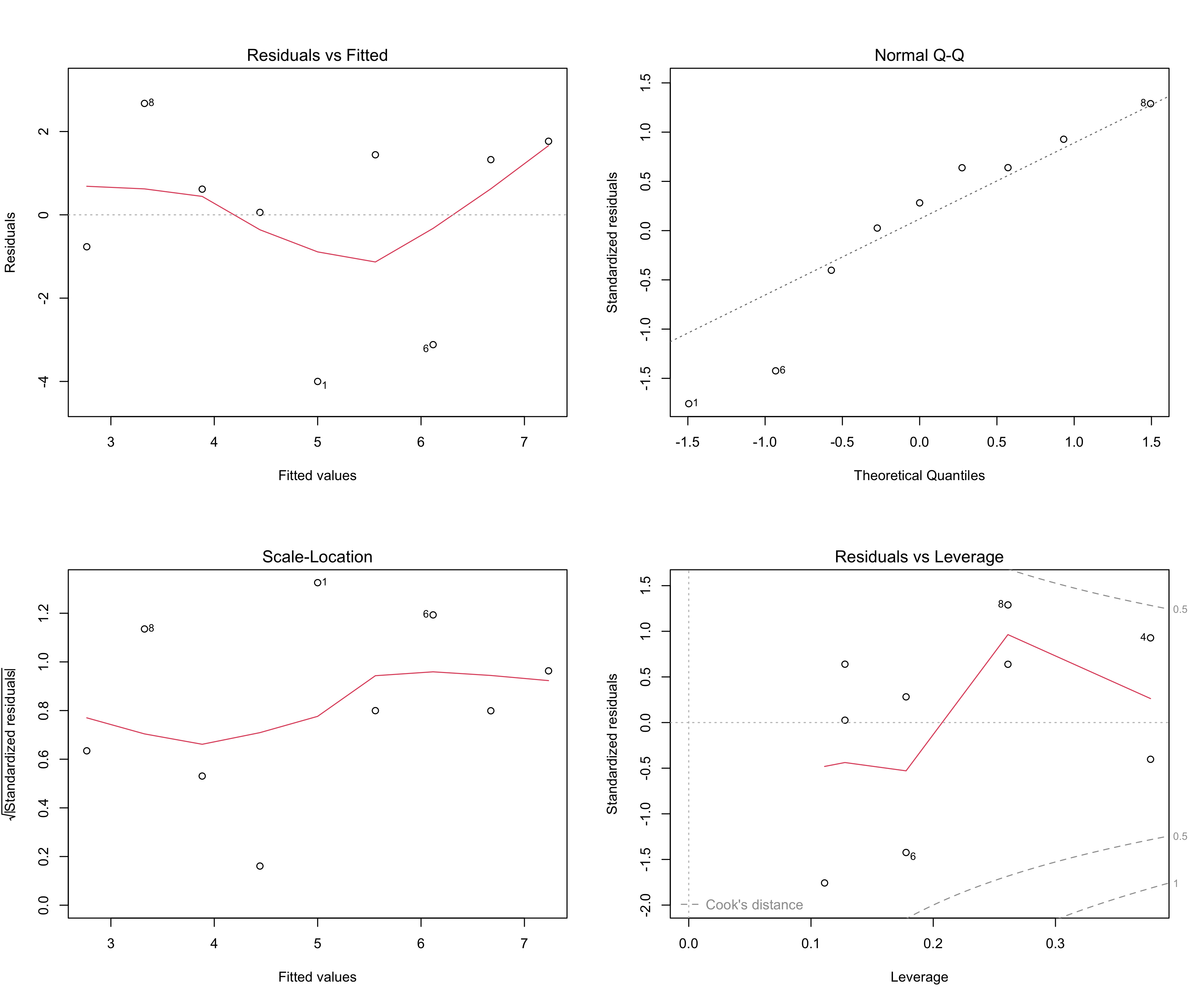


S11e – Residual analysis of *Pieris rapae*


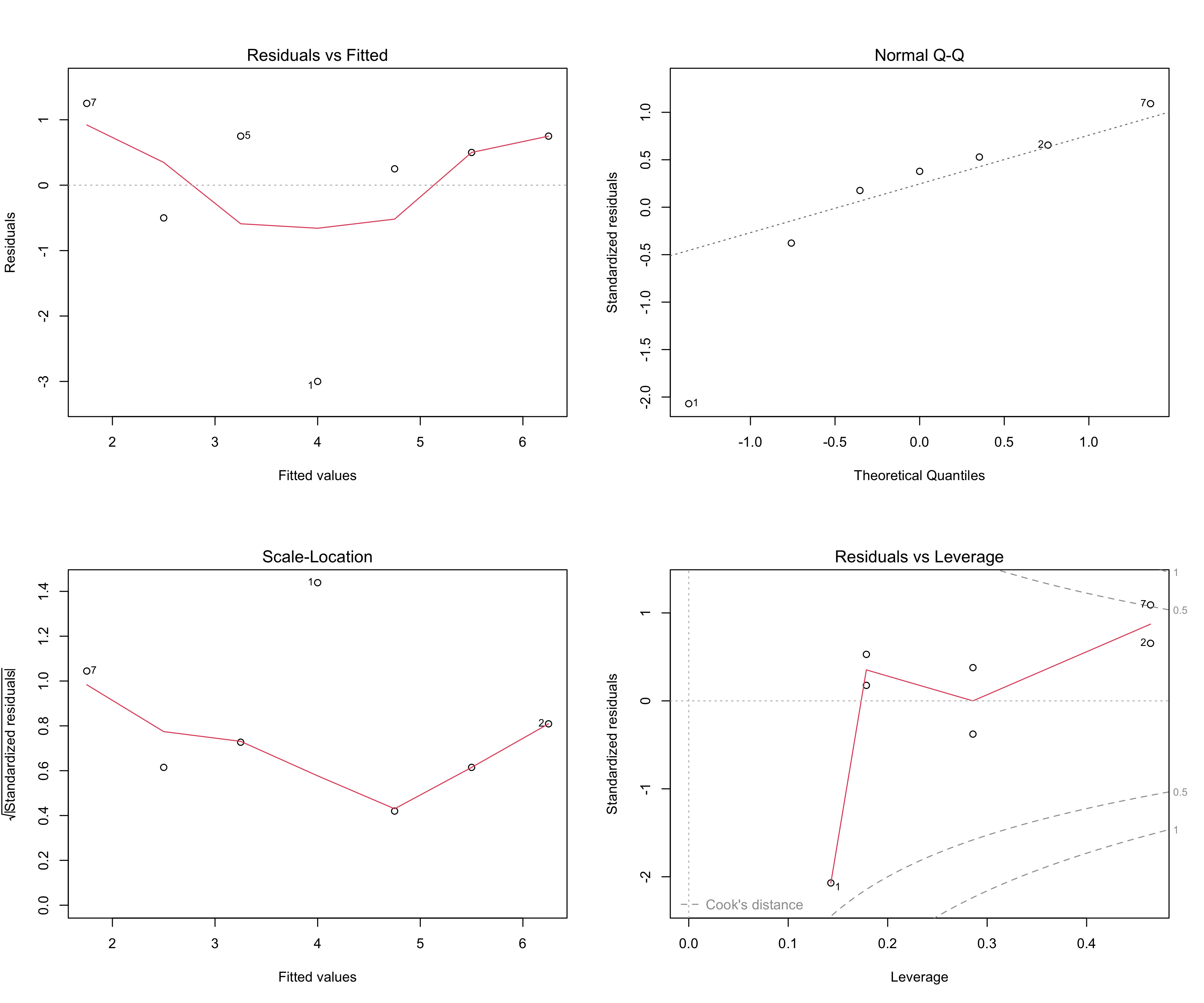


Tab. S4 – Confidence interval (95%) for slopes and intercepts of the regression equations reported in Figure S11 (*Coenonympha pamphilus,* Cp; *Lasiommata megera,* Lm*;* *Polyommatus icarus,* Pi*;* *Pieris napi,* Pn; *Pieris rapae,* Pr).

| Species | Confidence interval (95%) | | |
| --- | --- | --- | --- |
| Cp | Intercept | -2.18 | 4.47 |
|  | Slope | -0.03 | 1.46 |
| Lm | Intercept | -6.14 | 9.14 |
|  | Slope | -2.39 | 3.19 |
| Pi | Intercept | -2.03 | 5.03 |
|  | Slope | 0.07 | 1.33 |
| Pn | Intercept | -1.94 | 6.36 |
|  | Slope | -0.18 | 1.30 |
| Pr | Intercept | -2.40 | 4.40 |
|  | Slope | -0.01 | 1.51 |

Fig. S12 – Copper: bivariate Spearman’s correlation between soil and butterfly concentrations (mg kg^-1^ d.w.) (*Coenonympha pamphilus,* Cp; *Lasiommata megera,* Lm*;* *Polyommatus icarus,* Pi*;* *Pieris napi,* Pn; *Pieris rapae,* Pr).


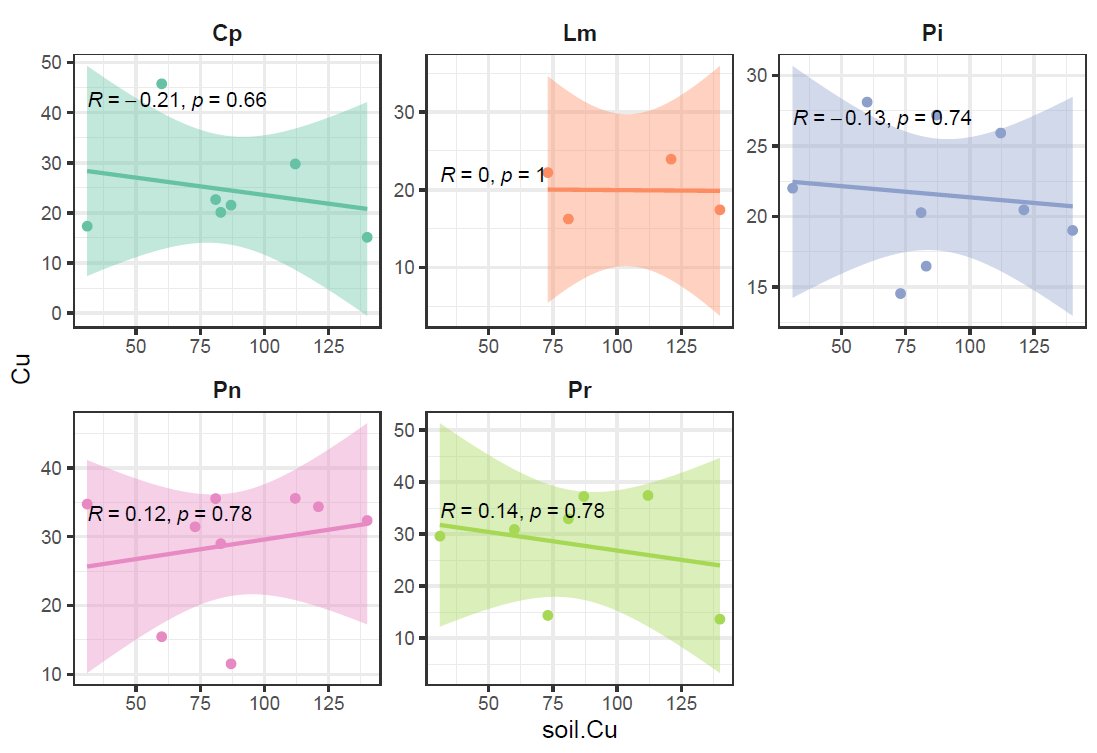


Fig. S13 – Iron: bivariate Spearman’s correlation between soil and butterfly concentrations (mg kg^-1^ d.w.) (*Coenonympha pamphilus,* Cp; *Lasiommata megera,* Lm*;* *Polyommatus icarus,* Pi*;* *Pieris napi,* Pn; *Pieris rapae,* Pr).


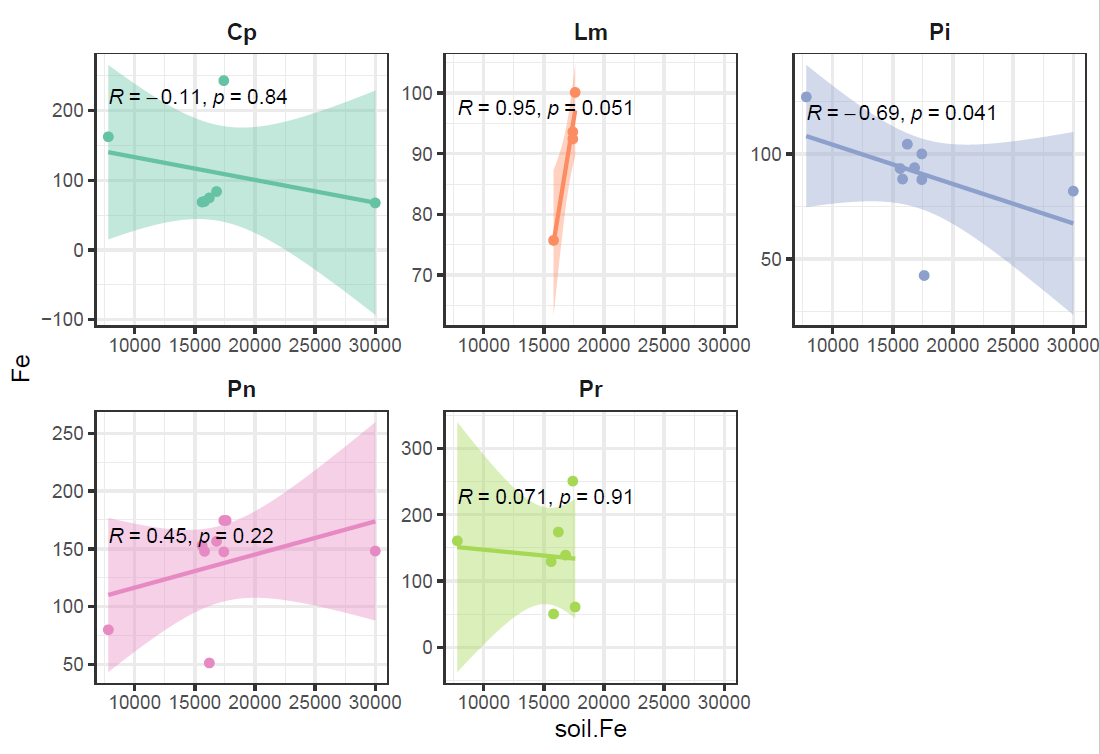


Fig. S14 – Manganese: bivariate Spearman’s correlation between soil and butterfly concentrations (mg kg^-1^ d.w.) (*Coenonympha pamphilus,* Cp; *Lasiommata megera,* Lm*;* *Polyommatus icarus,* Pi*;* *Pieris napi,* Pn; *Pieris rapae,* Pr).


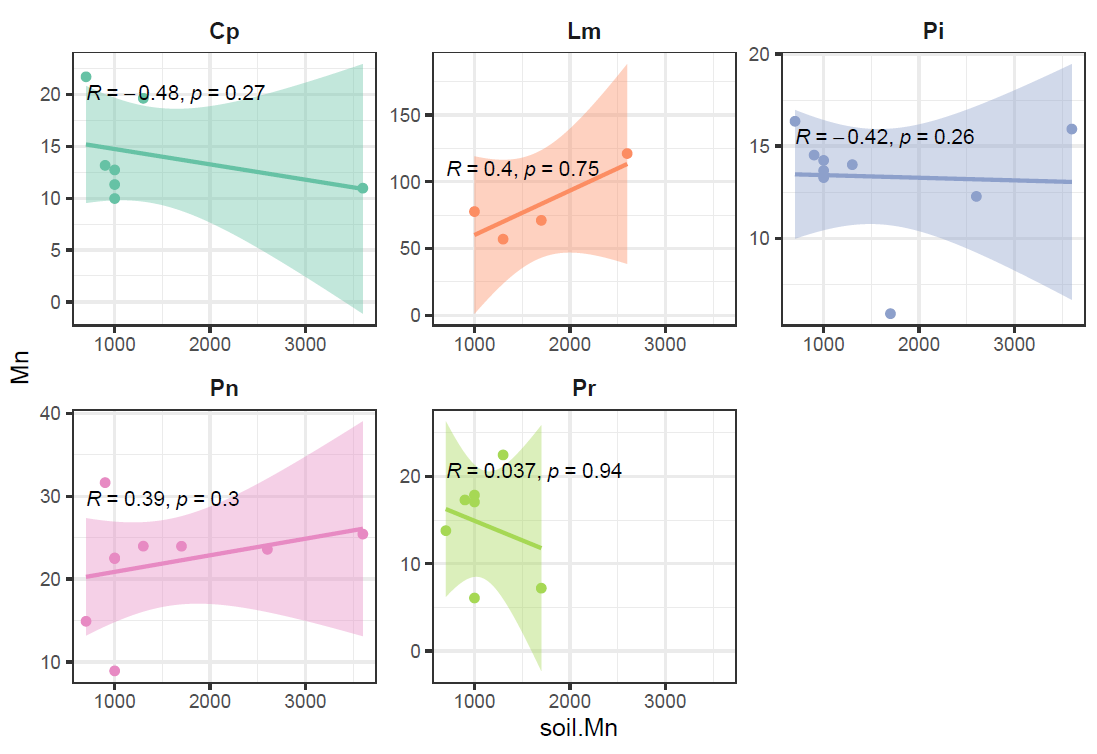


Fig. S15 – Nickel: bivariate Spearman’s correlation between soil and butterfly concentrations (mg kg^-1^ d.w.) (*Coenonympha pamphilus,* Cp; *Lasiommata megera,* Lm*;* *Polyommatus icarus,* Pi*;* *Pieris napi,* Pn; *Pieris rapae,* Pr).


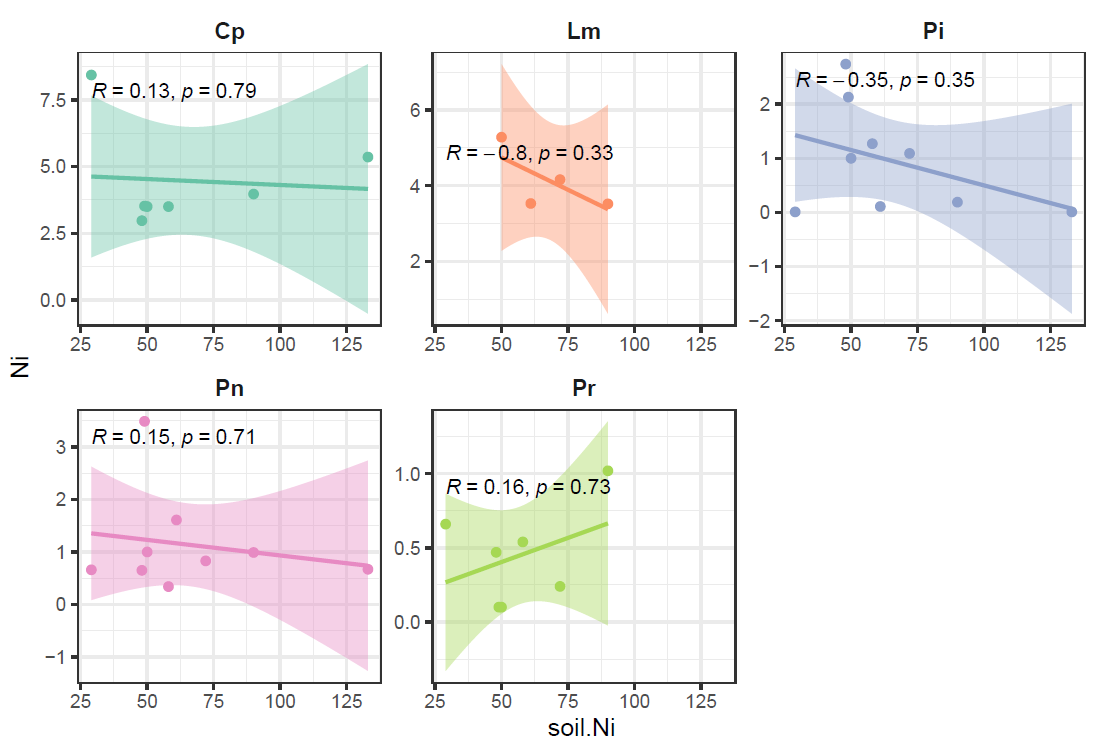


Fig. S16 – Lead: bivariate Spearman’s correlation between soil and butterfly concentrations (mg kg^-1^ d.w.) (*Coenonympha pamphilus,* Cp; *Lasiommata megera,* Lm*;* *Polyommatus icarus,* Pi*;* *Pieris napi,* Pn; *Pieris rapae,* Pr).


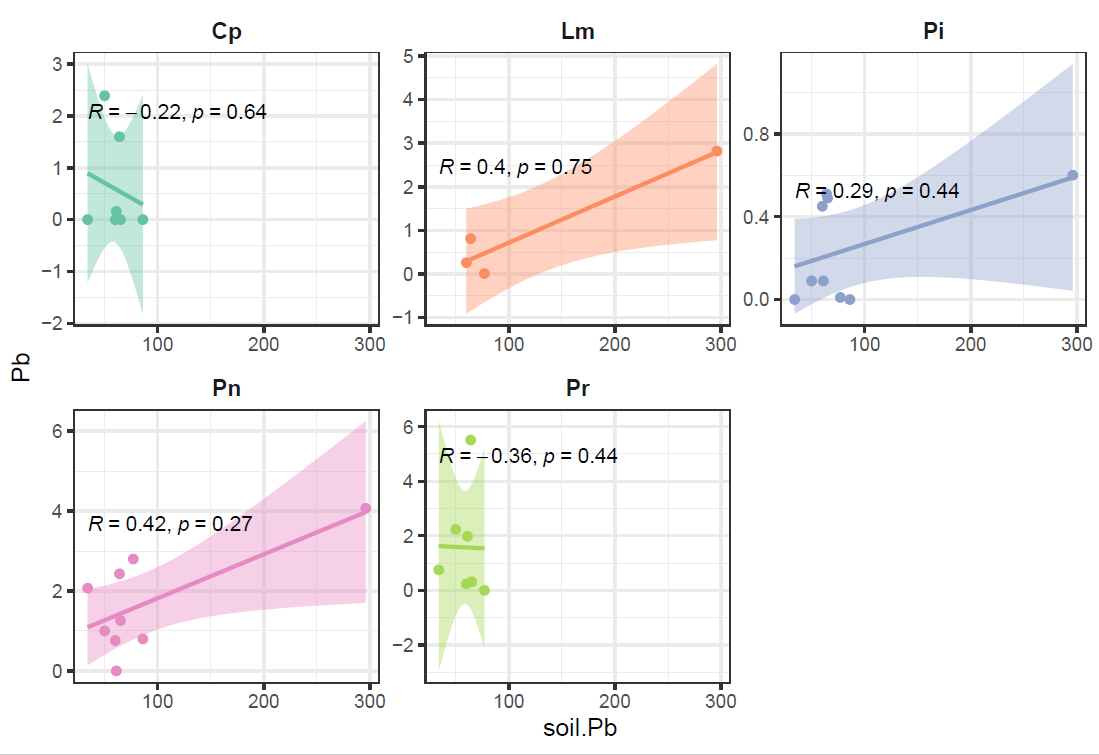


Fig. S17 – Strontium: bivariate Spearman’s correlation between soil and butterfly concentrations (mg kg^-1^ d.w.) (*Coenonympha pamphilus,* Cp; *Lasiommata megera,* Lm*;* *Polyommatus icarus,* Pi*;* *Pieris napi,* Pn; *Pieris rapae,* Pr).


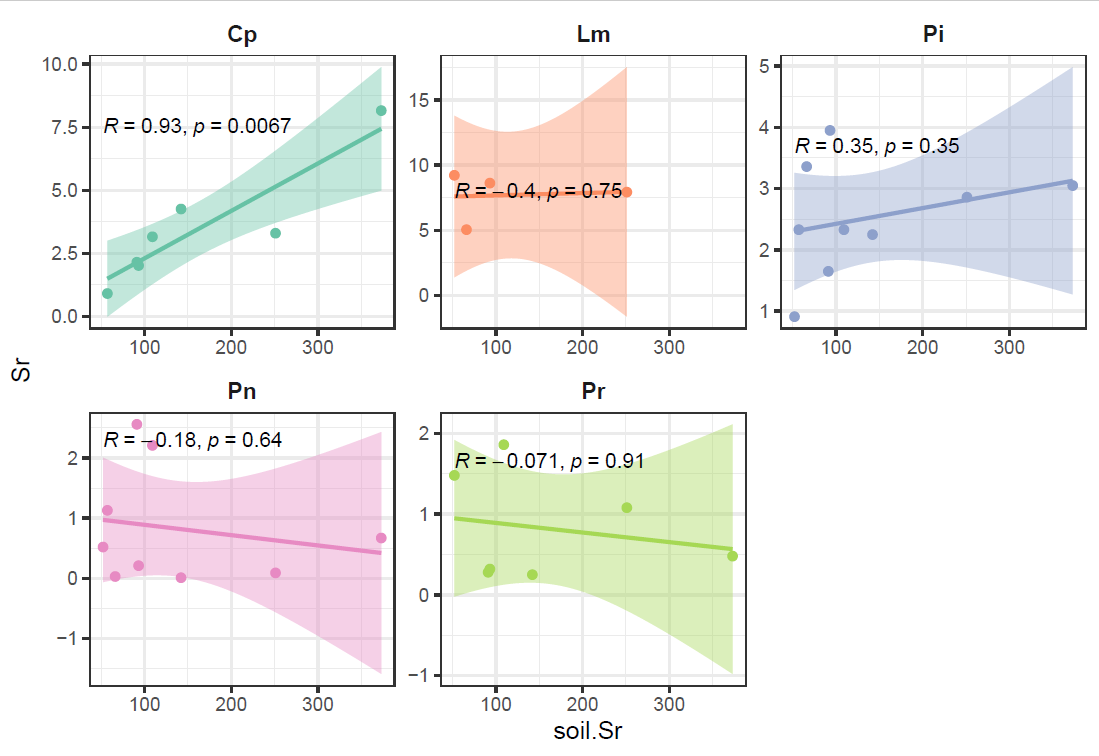


Fig. S18 – Zinc: bivariate Spearman’s correlation between soil and butterfly concentrations (mg kg^-1^ d.w.) (*Coenonympha pamphilus,* Cp; *Lasiommata megera,* Lm*;* *Polyommatus icarus,* Pi*;* *Pieris napi,* Pn; *Pieris rapae,* Pr).


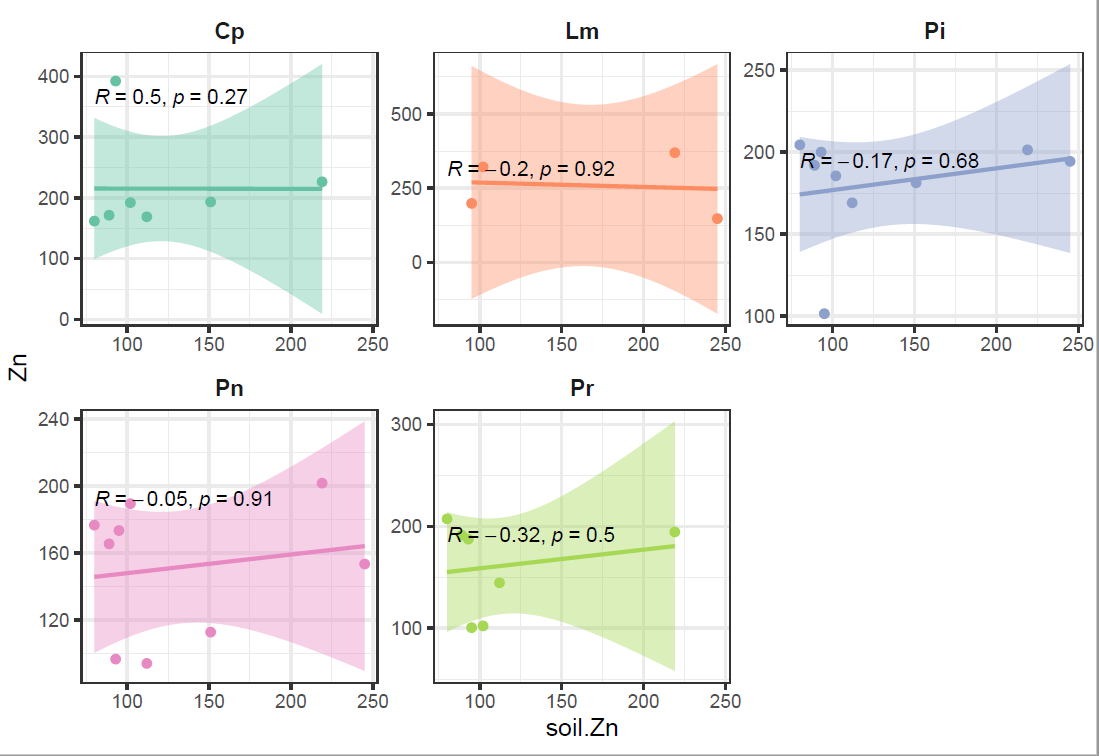

Supplement: Supplementary file 1 — Supplementary file1 (DOCX 3528 KB) [file 11356_2023_28930_MOESM1_ESM.docx]
